# Development and application of a high-throughput screening assay for identification of small molecule inhibitors of the *P. falciparum* reticulocyte binding-like homologue 5 protein

**DOI:** 10.1016/j.ijpddr.2020.10.008

**Published:** 2020-10-29

**Authors:** Brad E. Sleebs, Kate E. Jarman, Sonja Frolich, Wilson Wong, Julie Healer, Weiwen Dai, Isabelle S. Lucet, Danny W. Wilson, Alan F. Cowman

**Affiliations:** a The Walter and Eliza Hall Institute of Medical Research, Parkville, Victoria, 3052, Australia; b Department of Medical Biology, The University of Melbourne, Parkville, Victoria, 3052, Australia; c Research Centre for Infectious Diseases, School of Biological Sciences, University of Adelaide, Adelaide, South Australia, 5005, Australia

**Keywords:** *Plasmodium falciparum*, Erythrocyte invasion, Reticulocyte binding-like homologue 5, Drug screening

## Abstract

The *P. falciparum* parasite, responsible for the disease in humans known as malaria, must invade erythrocytes to provide an environment for self-replication and survival. For invasion to occur, the parasite must engage several ligands on the host erythrocyte surface to enable adhesion, tight junction formation and entry. Critical interactions include binding of erythrocyte binding-like ligands and reticulocyte binding-like homologues (Rhs) to the surface of the host erythrocyte. The reticulocyte binding-like homologue 5 (Rh5) is the only member of this family that is essential for invasion and it binds to the basigin host receptor. The essential nature of Rh5 makes it an important vaccine target, however to date, Rh5 has not been targeted by small molecule intervention. Here, we describe the development of a high-throughput screening assay to identify small molecules which interfere with the Rh5-basigin interaction. To validate the utility of this assay we screened a known drug library and the Medicines for Malaria Box and demonstrated the reproducibility and robustness of the assay for high-throughput screening purposes. The screen of the known drug library identified the known leukotriene antagonist, pranlukast. We used pranlukast as a model inhibitor in a post screening evaluation cascade. We procured and synthesised analogues of pranlukast to assist in the hit confirmation process and show which structural moieties of pranlukast attenuate the Rh5 – basigin interaction. Evaluation of pranlukast analogues against *P. falciparum* in a viability assay and a schizont rupture assay show the parasite activity was not consistent with the biochemical inhibition of Rh5, questioning the developability of pranlukast as an antimalarial. The high-throughput assay developed from this work has the capacity to screen large collections of small molecules to discover inhibitors of *P. falciparum* Rh5 for future development of invasion inhibitory antimalarials.

## Highlights

• A high-throughput screening assay was developed to identify inhibitors of Rh5.
• The assay was applied in a screen of the MMV Malaria Box and a known drug library.
• Pranlukast was identified as a hit, but could not be conclusively validated.
• Assay enables future screens of large compound libraries to discover Rh5 inhibitors.

## Graphical abstract

**Figure:**
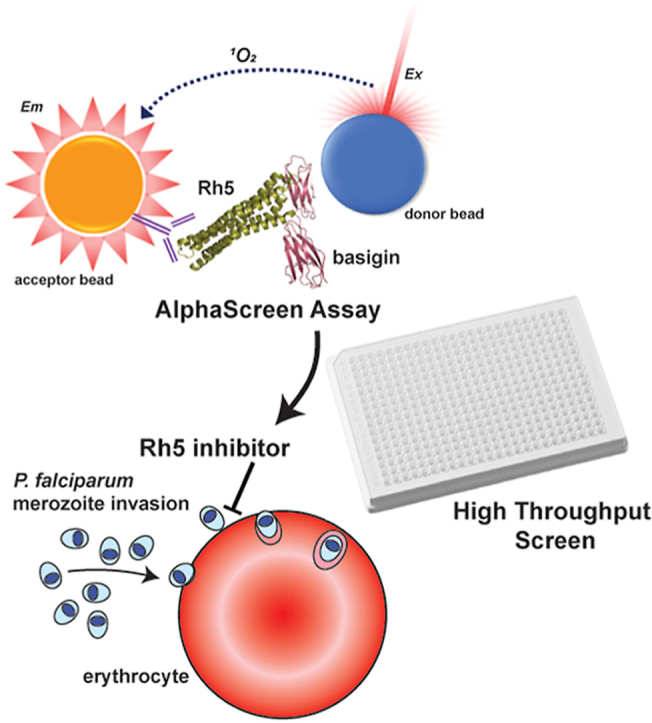

## 1 Introduction

Malaria is caused by infection with the genus of protozoan parasites known as *Plasmodium*. Each year these parasites cause approximately 228 million infections and 405,000 deaths. Of the five species that infect humans, *P. falciparum* and *P. vivax* are the most prevalent. *P. falciparum* is hyperendemic in Africa and is responsible for the most deaths globally. *P. vivax* is more endemic in South East Asia and is responsible for recrudescence of infection by activation of the dormant liver stage hypnozoite that reinitiates blood stage infection.

Current malaria control strategies include the use of antimalarial drugs, such as artemisinin combination therapy (ACT), and the use of insecticide treated bed nets to target the malaria mosquito vector. However, mounting drug-resistance in parasites, as well as widespread insecticide resistance in mosquitoes is threatening the efficacy of these control strategies. Recently, the first licensed vaccine (RTS,S) (trade name Mosquirix) was approved to protect against malaria, however it only offers limited protection for certain cohorts of the population (Bejon et al., 2013; RTS, 2012). Currently, there are a number of promising small molecule candidates undergoing preclinical and clinical phase assessment from the world antimalarial therapeutic portfolio (Ashton et al., 2019). Concerningly, a number of these candidates have a low barrier to resistance, and therefore it is essential that novel candidates are developed to populate the antimalarial clinical pipeline.

To survive the *Plasmodium* parasite must invade and reside within the host erythrocyte. Here, the parasite remodels the host erythrocyte to create an environment to replicate and to evade the host immune system (Mbengue et al., 2012). The invasion process begins when the merozoite form of the parasite recognises and adheres to receptors on the surface of the red blood cell (RBC). The merozoite then re-orientates itself, so the apical tip of the parasite is juxtaposed to the RBC. This aligns the rhoptry organelles with the surface of the RBC enabling the release of parasite proteins essential for invasion and positions the merozoite to form a tight junction. The merozoite then drives itself into the RBC membrane using its actin-myosin motor, and in the process, initialises the formation of the parasitophorous vacuole (Cowman et al., 2012; Weiss et al., 2015). On completion of invasion, the parasitophorous vacuole completely surrounds the merozoite and provides a secure environment for remodeling and exploiting the host RBC (Mbengue et al., 2012).

For the *P. falciparum* parasite to invade the RBC, a number of intimate interactions with the surface of the merozoite and the RBC take place (reviewed in (Counihan et al., 2013)). One key interaction is mediated by the conserved *P. falciparum* protein reticulocyte binding-like homologue 5 (Rh5) with the host erythrocyte receptor basigin (Crosnier et al., 2011). Rh5 is secreted from apical organelles upon invasion and is believed to be secured to the merozoite membrane and interacts with *P. falciparum* Rh5-interacting protein (Ripr) (Chen et al., 2011) and the cysteine-rich protective antigen (CyRPA) (Reddy et al., 2015; Volz et al., 2016). Rh5 is refractory to genetic deletion and is known to be essential for invasion and pathogenesis of the *P. falciparum* parasite (Baum et al., 2009; Crosnier et al., 2011; Hayton et al., 2008). Rh5 forms a complex by binding to CyRPA which then interacts with the Rh5-binding interacting protein (Ripr) (Chen et al., 2014; Reddy et al., 2015). The Ripr/CyRPA/Rh5-basigin complex is essential for establishing the tight junction and the subsequent sequential molecular events leading to parasite invasion of the erythrocyte (Volz et al., 2016). This complex binds efficiently to basigin and 3-dimensional changes in Rh5 are involved in insertion of part of this complex into the erythrocyte membrane during invasion (Wong et al., 2018). Given the importance of Rh5 in *P. falciparum* survival, Rh5 it is currently under investigation as a novel blood-stage malaria vaccine candidate (Drew and Beeson, 2015). Recent data has demonstrated that antibodies to Rh5 block *P. falciparum* invasion of the erythrocyte *in vitro* (Alanine et al., 2019; Bustamante et al., 2013; Chiu et al., 2014; Healer et al., 2019) and more recent data has shown Rh5-based vaccines can protect *Aotus* monkeys when challenged with a *P. falciparum* infection (Douglas et al., 2015). Clinical trials in healthy volunteers are currently in progress to assess tolerability and immunological response to a Rh5-based vaccine (Payne et al., 2017; Ragotte et al., 2020).

To the best of our knowledge, the Rh5 – basigin interaction has not been targeted with small molecules. We reasoned that small molecule inhibitors of Rh5 would block merozoite entry into the RBC and in turn kill the *P. falciparum* parasite. The Rh5 – basigin interaction is a protein-protein interaction (PPI) and historically in drug discovery these types of interactions have been notoriously difficult to target with small molecule therapies. Nevertheless, examples of PPI inhibitors in literature exist, such as ABT-199 (Venetoclax) (Souers et al., 2013) and ABT-263 (Navitoclax) (Park et al., 2008), inhibitors of the B-cell lymphoma 2 (BCL-2) family of proteins. These compounds were discovered using a fragment-based drug discovery approach. More recently, WEHI-539 a selective inhibitor of B-cell lymphoma-extra large (BCL-XL) was discovered using a high-throughput screening approach (Lessene et al., 2013) supporting the feasibility of our screening approach to identify inhibitors of the Rh5 – basigin PPI.

Herein, we describe the development of an assay using AlphaScreen (amplified luminescent proximity homogeneous assay screen) technology for implentation in high throughput screens of large compound libraries to identify inhibitors of the Rh5 – basigin interaction. To validate the assay for high-throughput (HT) screening purposes, we opted to screen a known drug library and the Medicines for Malaria (MMV) Malaria Box (Spangenberg et al., 2013). The known drug library comprises a set of 3707 compounds that are on-market or clinically used drugs, collated from the Tocris, LOPAC and Prestwick commercial vendor libraries. The MMV Malaria Box (Spangenberg et al., 2013) is a selection of 400 compounds from larger screening hit sets that possess antimalarial activity. The Malaria Box was derived from 20,000 hits obtained from screening 4 million compounds from the libraries of St Jude Children's Research hospital (Guiguemde et al., 2010), Novartis (Gagaring et al.), GSK (Gamo et al., 2010) against the asexual stage of *P. falciparum* parasites. The reproducibility and robustness statistics derived from the screen will be used to demonstrate the application of the assay in the future HT screens of large compound libraries to identify compounds that bind to Rh5 and have properties suitable for development of an antimalarial that blocks merozoite invasion of the erythrocyte.

## 2 Materials and methods

### 2.1 Recombinant protein expression and purification, and antibody production

Recombinant *P. falciparum* (3D7) Rh5 was expressed and purified according to the previously described procedure of Chen et al. (2014). Briefly, a synthetic gene encoding *P. falciparum* (3D7) full-length mature PfRh5 (residues 24–526) was inserted into insect/mammalian cell expression vector pgpHFT (Xu et al., 2010) using *Kpn* I and *Xho* I sites to produce pgpHFT-PfRh5. The pgpHFT-PfRh5 was then co-transfected with FlashBAC (Oxford Expression Technologies) into Sf21 insect cells as per supplier's manual. The seed virus was amplified to obtain high-titer viral stocks, which were then used to infect Hi5 cells grown in express Five SFM medium (Life Technologies Pty Ltd, Australia) supplemented with 1 mM glutamine. The supernatant containing the secreted recombinant protein was harvested, centrifuged, and passed over anti-FLAG M2 agarose (Sigma-Aldrich, Australia) column. After extensive washing, bound proteins were eluted from the column with the FLAG peptide at a concentration of 100 μg/mL, concentrated and further purified by size-exclusion chromatography with a Superdex 200 column (GL 10/300, GE Healthcare, Australia) in 50 mM Tris, 100 mM NaCl, pH 8.5.

Human basigin isoform 2 was expressed in insect cells and purified as described previously (Crosnier et al., 2011). The Rh5 mouse monoclonal antibody was prepared according to the previously described procedure of Baum et al. (2009). The basigin monoclonal antibody was recombinantly expressed using a previously described procedure (Crosnier et al., 2011).

Doublecortin-like kinase domain 1 (DCLK1) used as a control protein in differential scanning fluorimetry experiments and was recombinantly expressed and purified according to the previously described protocol (Patel et al., 2016).

### 2.2 Rh5-basigin AlphaScreen and counterscreen assay

Screening of the compounds was performed using the AlphaScreen detection kit system (PerkinElmer Lifesciences). The assay contains the following regents, Rh5 protein (50 nM final concentration), hexa-histidine tagged basigin (20 nM final concentration), anti-Rh5 mouse monoclonal antibody (1 μg/mL final concentration), Protein A-coated acceptor beads (final concentration of 10 μg/mL) (PerkinElmer 6760137M) and nickel chelate-coated donor beads (final concentration of 10 μg/mL) (PerkinElmer 6760002B). The assay buffer (pH 7.4) contained 25 mM HEPES, 25 mM Tris, 50 mM NaCl, 0.005% Tween 20, and 0.1 mg/mL casein. The final DMSO concentration in the assay was 1.0% (v/v).

A compound master plate was prepared using 384-well low volume plates (Corning #3672) containing either fixed concentration of compounds in DMSO for the high-throughput screen of the compound libraries (final compound concentration of 20 μM) and confirmation (final compound concentration of 10 μM) or for compound IC_50_ determination compounds were serially diluted (2-fold, top final concentration of 50 μM) with DMSO in a 11 pt titration (in duplicate) from a 10 mM DMSO stock solution of compounds. Using the Combi-multidrop, 5 μL of Rh5 stock solution (200 nM prepared in assay buffer) was added to columns 1–24 of the 384-well assay plate (Greiner 384-well white, low binding plate, #784904, Bioone). The PerkinElmer Janus automated dispensing system was then employed to transfer, via the pin-tool, 2 × 100 nL of the compound stock DMSO solution from the compound master plate to the assay plate. The plate was sealed and incubated for 30 min at room temperature. Using a Combi-multidrop, 5 μL of hexa-histidine tagged basigin stock solution (80 nM prepared in assay buffer) was then added to columns 1–23 and 5 μL assay buffer to column 24 of the 384-well assay plate. The plate was sealed again and incubated for 30 min at room temperature. A solution of AlphaScreen donor and acceptor beads (20 μg/mL prepared in assay buffer) plus anti-Rh5 mAb solution (2 μg/mL prepared in assay buffer) was prepared in assay buffer under subdued lighting. Using the Combi-multidrop, 10 μL of this solution was added to every well of the assay plate under subdued lighting. The plate was sealed with foil (plate max) and incubated at room temperature for 1 h. The assay plate was then analysed on the PerkinElmer Envision 2103 Multilabel Plate Reader (Ex 680, Em 520–620 nm).

The counterscreen assay was conducted using AlphaScreen TruHits kit (PerkinElmer). Biotinylated acceptor beads and Streptavidin donor beads were both diluted in assay buffer (final concentration of 10 μg/mL). Using the Combi-multidrop, 10 μL of each bead solution (20 μg/mL prepared in assay buffer) was added to every well of the assay plate under subdued lighting. Compound addition and assay readout was performed as described above.

For the high-throughput screen of compound libraries, data was uploaded and analyzed using ABase and Tibco Spotfire software. For the IC_50_ determination of compounds, IC_50_ values of compounds were calculated using either ABase or GraphPad Prism (version 6.05) software. A nonlinear regression four-parameter fit analysis was undertaken in which the parameters were not constrained. The equation used is sigmoidal dose response (variable slope), Y = bottom + (top − bottom)/(1 + 10((logEC_50_ − X) × Hill Slope)).

### 2.3 Compound synthesis and procurement

A similar preparation to that by Raposo et al. (1996) and Green et al. (Geen et al., 2006) was followed for synthesis of pranlukast analogues. A detailed description of compound synthesis and compound characterisation can be found in the associated supporting information section. Pranlukast, zafirlukast and montelukast were purchased from AK-Scientific. Cinalukast, FPL 55712, SR 2640, and MK 571 were purchased from Tocris.

### 2.4 Differential scanning fluorimetry assay

Differential scanning fluorimetry assays were performed as described previously (Murphy et al., 2013, 2014a, 2014b) using a Rotor-Gene Q PCR. Briefly, purified PfRh5 was diluted in 20 mM Tris pH 8.0, 150 mM NaCl to achieve 10 μg per reaction and assayed with the appropriate concentration of inhibitor in a total reaction volume of 25 μL. SYPRO Orange (Molecular Probes, CA) was used as a fluorescence probe and detected at 530 nm. Compounds were titrated at final concentrations ranging from 5 μM to 80 μM final. Data was transformed and plotted using GraphPad Prism. Shown data are representative means of two technical experiments.

### 2.5 *P. falciparum* parasite viability assay

A modified version of the lactate dehydrogenase (LDH) assay described by Gamo et al. (2010) was used to assess the activity of compounds against asexual *P. falciparum* 3D7 parasites. Compounds were pre-dispensed in 384-well plates, RPMI/Albumax growth media was added, and *P. falciparum* inoculated. Plates were incubated for 72 h and then frozen at −80 °C overnight. LDH activity was quantified with the modified cofactor 3-acetylpyridine adenine dinucleotide (APAD) (Sigma Aldrich) by measuring absorbance of the tetrazolium indicator nitro blue tetrazolium (NBT) (Sigma Aldrich) at 650 nm.

Parasite conditions: An inoculum of synchronous *P. falciparum* (3D7 strain) parasitised red blood cells (PRBC) at 0.7% parasitaemia and 2% haematocrit in RPMI-1640, 5% Albumax, 2% D-sucrose, 0.3% glutamine and 150 μM hypoxanthine was used for the assay.

Growth inhibition assay: Compound master plates (384-well) were prepared by a 10 pt serial dilution of compounds, from 10 mM to 98 μM, in columns 3–12 and 13–22. DMSO was dispensed into columns 1 and 23 of the compound master plate to be used as the positive growth control (100% viability). Columns 2 and 24 of the compound master plate had a stock concentration of 200 μM chloroquine solution (0% viability) as negative growth control (final assay concentration of 200 nM). Intermediate compound dilution plates were prepared by dispensing 5 μL from each well of the compound master plate into 11.5 μL of RPMI/Albumax growth media. Duplicate assay plates (384-well) were then prepared by dispensing 0.5 μL of compound from the intermediate dilution plates into 9.5 μL of RPMI/Albumax growth media. The parasite inoculum (30 μL) was dispensed into the assay plates containing compounds using a Multidrop dispenser (Thermo Scientific) such that the final assay volume was 40 μL and final compound concentration was 50 μM–98 nM (the volume of compound addition can be adjusted to the preferred and agreed screening concentration). The final DMSO concentration was 0.1% (to limit toxicity to parasites). Plates were incubated at 37 °C for 72 h in an atmosphere of 5% CO_2_, 5% O_2_, 95% N_2_.

Evaluation of parasite growth measuring lactate dehydrogenase (LDH) activity: After 72 h of incubation, plates were frozen at −80 °C overnight and then thawed at room temperature for at least 4 h. To evaluate LDH activity, 45 μL of freshly made reaction mix (174 mM sodium L-lactate (Sigma Aldrich), 214 μM 3-acetyl pyridine adenine dinucleotide (APAD) (Sigma Aldrich), 270 μM nitro blue tetrazolium chloride (NBT) (Sigma Aldrich), 4.35 U/mL diaphorase (from *Clostridium kluyveri*) (Sigma Aldrich), 0.7% Tween 20, 100 mM Tris-HCl pH 7.5) was dispensed using a Multidrop dispenser (Thermo Scientific). Plates were shaken to ensure mixing and absorbance at 650 nm was monitored using a PerkinElmer Envision plate reader after 30 min of incubation at room temperature. Data were normalised to percent growth inhibition using positive and negative controls and analyzed using TIBCO Spotfire software.

### 2.6 Schizont rupture assay

D10-PfPHG parasites (Wilson et al., 2010) were cultured in human O^+^ erythrocytes in RPMI-HEPES culture medium (pH 7.4, 50 μg/mL hypoxanthine, 25 mM NaHCO_3_, 20 μg/mL gentamicin, 0.5% Albumax II (Thermo Fisher Scientific) according to established protocols (Trager and Jensen, 1976) and maintained in an atmosphere of 1% O_2_, 4% CO_2_ and 95% N_2_. For assessment of merozoite invasion after schizont rupture, parasites were tightly synchronised to a 4 h window of invasion using heparin (Boyle et al., 2010; Wilson et al., 2015) and then grown for a further 42 h. Drug treatments were setup at 2–3% parasitaemia and 1% haematocrit in 50 μL volumes with a 1 in 10 dilution of drug added when parasites were 44–48 h post invasion. After 6 h of drug treatment and immediately after invasion was expected to finish, the assays were treated with 5 μg/mL ethidium bromide (EtBr, Bio-Rad) for 5 min prior to flow cytometry assessment of parasitaemia using a Becton Dickinson Accuri. Gating of newly invaded rings, free merozoites and unruptured late stages was achieved as per published methods (Wilson et al., 2013).

### 2.7 HepG2 growth inhibition assay

HepG2 cells were cultured in Dulbeccos modified eagle's medium (DMEM) supplemented with 10% fetal calf serum (FCS), in a humidified incubator at 37 °C and 5% CO_2_. Ten-point compound titration assays were performed by treating cells (1 × 10^3^) for 48 h in 384 well tissue culture treated plates (Greiner). Cytotoxicity was determined using Cell Titer Glo (Promega) and calculated as a percentage using DMSO as a positive growth control and 10 μM Bortezomib as a negative growth control. EC_50_ values were calculated using a 4-parameter log dose, non-linear regression analysis, with sigmoidal dose response (variable slope) curve fit using Graph Pad Prism (version 6.05). 0 and 100 constraint parameters were used for curve fitting. Etoposide was used as a control compound and was determined to have an EC_50_ of 15.9 μΜ, compared to the literature value EC_50_ of 30.2 μΜ that had an incubation time of 48 h using an MTT assay to determine cell viability (Pingaew et al., 2014).

## 3 Results

### 3.1 Development of the high-throughput screening assay

To identify inhibitors of the Rh5 we developed an assay using ALPHA (amplified luminescent proximity homogeneous assay) screen technology. The decision to use the AlphaScreen assay format was founded on our past experience using this assay format with discovering inhibitors of PPIs (Lessene et al., 2013).

In the development of the assay, we used an anti-Rh5 mouse monoclonal antibody (Rh5 mAb) to append Rh5 to the protein A acceptor beads obtained from PerkinElmer (Fig. S1). The production of the Rh5 mAb and Rh5 were produced as previously described (Baum et al., 2009; Chen et al., 2014). Hexa-histidine tagged basigin was produced following the protocol of Crosnier et al. (2011), and was captured on Ni^2+^ chelate donor beads acquired from PerkinElmer. To develop an assay suitable for the purposes of high-throughput screening, we first set out to optimise the AlphaScreen signal using the Rh5 bound acceptor beads and the basigin bound donor beads. In this optimisation process, the quantities of both donor and acceptor beads recommended by PerkinElmer were used and initially 2 μg/mL of the Rh5 mAb. The assay was buffered at pH 7.4 and the non-ionic surfactant, Tween 20, was added for liquid handling purposes. Casein was also added in addition to Tween 20 to prevent promiscuous aggregation of proteins or compounds, that can affect assay performance, limiting false-positive hits.

To begin the assay optimisation, we assessed the quantities of both Rh5 and basigin required to maximise the AlphaScreen signal compared to the background. To do this, both Rh5 and basigin were titrated in a diluted series to measure the effect on the AlphaScreen signal. The results of this titration are plotted in Fig. S2 (panel A and B) and show the classic ‘hook’ standard curve observed with dilution series in AlphaScreen assays (Yasgar et al., 2016). The hook effect is observed when a high concentration of one binding partner saturates the interacting partner protein, and inhibits their interaction resulting in a decrease in signal. The hook point or the plateau in the signal is the maximum concentration attained before saturation of the AlphaScreen beads. The selection of the optimal concentration of binding partners is normally the highest signal readout in the linear phase of the hook standard curve. From these results, the optimal concentration of Rh5 was 50 nM, and for basigin this concentration was approximately 12.5 nM. To find a more accurate optimal concentration of basigin, basigin was titrated at lower concentrations, while maintaining Rh5 at either 50 or 100 nM. The result of this titration shown in Fig. S2 (panel C and D), demonstrates that the optimal signal in the linear range is observed at a 20 nM concentration of basigin. We then measured the effect of varying the amount of Rh5 mAb from 2 μg/mL to 1 μg/mL in the Rh5 basigin titrations on the assay signal. The result of this experiment shown in Fig. S2 (panel E and F), demonstrates that an increased AlphaScreen signal is observed at 1 μg/mL of Rh5 mAb. The lower signal at higher antibody concentration of 2 μg/mL was likely observed due to the oversaturation of Rh5 mAb at the acceptor bead interface that resulted in sterically hindering the interaction between Rh5 and basigin. Thus, 1 μg/mL of Rh5 mAb was found to be optimal.

We next investigated whether a longer donor and acceptor bead incubation period would enhance the AlphaScreen signal. To this point of optimisation, a 1 h incubation period was used. To measure the effects of a longer incubation period, we used Rh5 and basigin at two concentrations and extended the time from 1 to 5 h. As seen in Fig. S2 (panel G and H), a longer incubation of donor and acceptor beads has no effect on the assay signal. Thus, 1 h was viewed as a suitable incubation time after the addition of the AlphaScreen donor and acceptor beads.

To validate the assay for the application of HT screening, we chose to screen a known drug library and MMV Malaria Box. The compounds for these screens were supplied as DMSO stock solutions and therefore compound dilutions/titrations were performed using DMSO in a 384-well master plate before transferring to the diluted/titrated compounds to the 384-well assay plate. The final concentration of DMSO in an assay is an important factor to consider in an AlphaScreen assay as DMSO is known to affect the assay signal. In addition, the final concentration of DMSO is also important to ensure effective transfer of compounds from master plate to the assay plate to attain the compound screening concentration of the assay. To assess the effect on DMSO concentration on the assay signal, a titration of DMSO was performed using the optimised assay conditions described earlier. The titration (Fig. S3) revealed that a final concentration of 2.5% of DMSO has minimal effect on assay signal. At concentrations above 2.5% the assay signal is significantly reduced. A final DMSO concentration of 2.5% was within the capacity limits of our automated liquid handling, and thus the primary screen of the compound libraries was conducted at 20 μM with a final DMSO concentration of 1%.

### 3.2 Screen of the known drug library and MMV malaria box

**Fig. 1:**
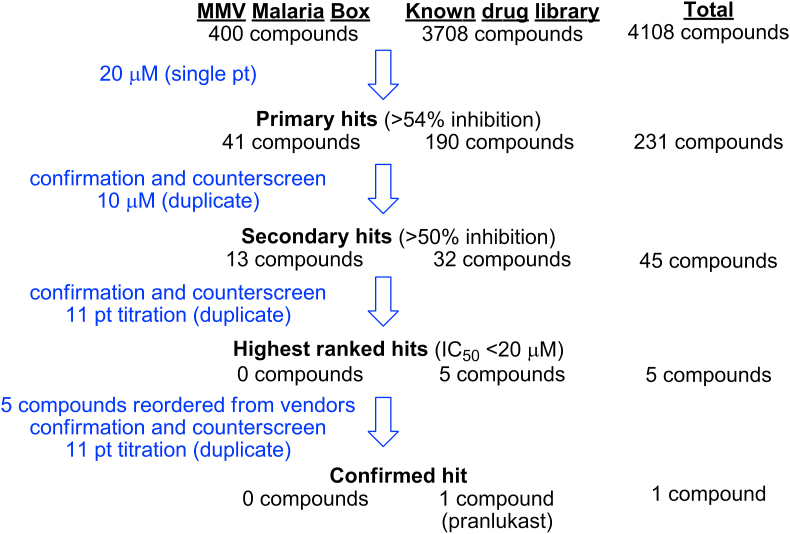
Overview of the cascade undertaken to screen the known drug library and the MMV Malaria Box against *P. falciparum* Rh5.

The optimised Rh5 AlphaScreen assay was then validated for HT screening purposes by conducting a pilot screen using known drug library (from Prestwick, Tocris and LOPAC) and the MMV Malaria Box, comprising 3708 and 400 compounds (Fig. 1), respectively. Historically, PPIs are notoriously difficult to target with small molecules. To enhance the likelihood of identifying an inhibitor of the Rh5 - basigin interaction the primary screen was conducted at a compound concentration of 20 μM. A 20 μM screening concentration is considered high, when compared to the commonly used primary screen concentrations of approximately 1–10 μM.

The primary pilot screen of the compound libraries (Table S1) was shown to be robust across all plates, as shown by the Z’ statistic of >8 for the known drug library plates and >7 for the Malaria Box plates (Fig. S4) and low signal-to-noise ratio (Fig. S5). The frequency of inhibition plot for both libraries screened (Fig. S6) was used to calculate and define a primary hit, commonly defined in HT screening as three standard deviations of the mean. To maximise the hit rate, two standard deviations of the mean was used, rather than the commonly used three standard deviations of the mean. Employing this calculation, a primary hit for the Malaria Box and the known drug libraries was defined as compounds that respectively displayed >57% and >54% inhibition in the AlphaScreen assay. This resulted in 41 primary hits from Malaria Box and 190 primary hits from the known drug libraries. The overall primary hit rate was 4.5%, which is considered high when compared to a hit rate of <1% that is usually observed for screens of compound libraries. The high primary hit was attributed to the high primary screening concentration, which is likely to result in a high number of false positive hits. Overall, the across plate robustness and reproducibility statistics (Fig. S4 – S6) obtained from the pilot primary screen demonstrated the utility of the assay for future HT screening of other compound libraries to identify inhibitors of Rh5.

To follow-up on hit compounds from the pilot validation screen, we next wanted to confirm the activity of the primary hits. To do this, a two-phase hit confirmation and counterscreen process was undertaken (Fig. 1). In the first phase, the 231 primary hits from both libraries were evaluated in the Rh5 AlphaScreen assay and counterscreen assay. Both assays were undertaken at 10 μM in duplicate. The counterscreen assay was conducted using AlphaScreen TruHits. The frequency of inhibition plot for both libraries (Fig. S7) and two standard deviations of the mean calculation was applied to the assay results, and secondary hits were defined as compounds that displayed >49% inhibition. The counterscreen cut-off of <40% inhibition was applied, and this resulted in 45 secondary hits. The 45 secondary hits can be viewed in the top left quadrant of Fig. S8. Of the 45 hits, 13 compounds originated from the Malaria Box and 32 compounds from the known drug library.

In the second phase of the confirmation pilot screening cascade, the 45 secondary hits were evaluated in the Rh5 AlphaScreen assay and the counterscreen assay in a 11 pt dose response format (top compound concentration of 50 μM in 2-fold dilution series) (Fig. 1). The results of these assays (Table S2 and Figs. S8 and S9) show that most of the 45 compounds were counterscreen positive in the 11 pt dose response format. From this process, compounds were ranked on potency in the Rh5 AlphaScreen assay and were discarded if they displayed activity in the counterscreen assay. We then selected the 5 highest ranked compounds and re-ordered these compounds from chemical vendors for re-evaluation. All the five compounds selected originated from the known drug libraries. The re-ordered solid samples of five highest ranked compounds were re-evaluated in the Rh5 AlphaScreen assay and the counterscreen assay in a 11 pt dose response format. The evaluation of the five re-ordered compound samples resulted in one confirmed hit, the cysteinyl leukotriene receptor antagonist, pranlukast (Fig. 1). In the Rh5 AlphaScreen assay, pranlukast exhibited an IC_50_ of 12 μM (Table 1). The four other re-ordered compound samples were not active in the AlphaScreen assay and therefore these four compounds, (*R*(-)-propylnorapomorphine, *R*(-)-2,10,11-trihydroxyaporphine, *R*(-)-apomorphine and rabeprazole) were excluded from further analysis. The confirmation and counter screen process eliminated false positive compounds and confirmed the activity of the hit compound, and therefore this process is suitable for follow-up of future high throughput screens against other compound collections to identify inhibitors of Rh5.

**Table 1:** Biological evaluation of pranlukast and analogues.

Compound	Rh5 IC_50_ μM^a^	*P. falciparum* 3D7 EC_50_ μM^b^	*P. falciparum* D10 EC_50_ μM^c^	HepG2 EC_50_ μM^d^	PSA (Å^2^)^e^	cLogP^e^
pranlukast	12	40	28	>50	116	4.4
zafirlukast	17	5.9	–	>50	113	6.4
FPL 55712	28	>50	–	>50	122	4.5
SR2540	>50	–	6.1	>50	74	5.7
cinalukast	>50	–	32	>50	82	5.5
**11**	>50	–	–	–	100	4.7
**12**	>50	–	–	–	100	4.6
**13**	>50	>50	>50	>50	91	5.4
**14**	>50	–	–	–	91	5.9
**15**	>50	–	–	–	105	4.0
**16**	40	37	6.7	>50	105	4.2
**17**	24	15	19	>50	105	4.7
**18**	21	>50	42	>50	105	5.1
**20**	>50	–	–	–	91	3.0
**21**	>50	>50	–	>50	105	2.2
**24**	>50	25	–	>50	116	1.8
**25**	20	7.3	–	>50	116	4.3

a An 11-point dilution series of each compound was incubated (20 °C) with Rh5 and basigin. IC_50_ data represents means for three independent biological replicates in the AlphaScreen assay. ±SEM < 5.5 (except for **17** and **24** with ±SEM of 7.7 and 9.5 respectively); dose response variation shown in Fig. S10.

b EC_50_ data represents means for three independent biological replicates measuring LDH activity of *P. falciparum* 3D7 parasites following exposure to compounds in a 10-point dilution series for 72 h. SD < 5 μM; dose response variation shown in Fig. S13. Controls: Brefeldin A EC_50_ 3.5 μM; Chloroquine EC_50_ 23 nM.

c EC_50_ data represents means for 3 independent biological replicates measuring *P. falciparum* D10 growth inhibition using flow cytometry following exposure to compounds in a 12-point dilution series for 72 h. SD < 7 μM (except for **13** with SD of 13); dose response variation shown in Fig. S14.

d EC_50_ data represents means for 3 technical replicates of the HepG2 growth inhibition assay in a 10-point dilution series over 48 h. Cell Titre-Glo was used to quantify cell growth inhibition.

e Physicochemical properties calculated using ChemAxon software (Calculator Plugins were used for structure property prediction and calculation. MarvinSketch 6.0.6.).

### 3.3 Differential scanning fluorimetry evaluation of hit binding to Rh5

**Fig. 2:**
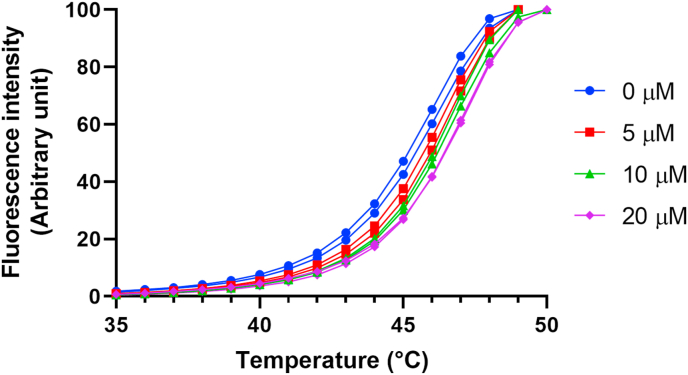
Differential scanning fluorimetry analysis of Rh5 using pranlukast at concentrations of 0–20 μM showing stabilisation of the Rh5 protein. Data shown represents two independent experiments.

Differential scanning fluorimetry (DSF) or thermal shift analysis is one method that could be used to confirm hits are binding to Rh5 in future HT screens. We used this technique to help confirm the Rh5 binding activity of the pilot screen hit compound, pranlukast. Additionally, these studies could be used to demonstrate that the hit was binding to Rh5 and not basigin. In this study, purified Rh5 displays a melting temperature (Tm) of 46 °C. We conducted a titration of pranlukast at concentrations consistent with the IC_50_ value determined against Rh5 in the AlphaScreen assay (Table 1). The results show that pranlukast at concentrations of 0–20 μM resulted in a dose dependent increase in the Tm of Rh5 (Fig. 2), which is indicative of pranlukast binding to and stabilising Rh5. To ensure the interaction with Rh5 by pranlukast was not promiscuous, we employed doublecortin like kinase 1 (DLCK1) (Patel et al., 2016) as an arbitrary control protein that was available in our laboratories. The results demonstrate that pranlukast at concentration up to 20 μM did not have a significant impact on the Tm of DCLK1 (Fig. S11), implying pranlukast did not non-specifically bind to Rh5. We could not use basigin as a control protein in DSF analysis, because basigin was prepared in a buffer that contains a detergent that is not compatible with the DSF technique. Nevertheless, the data collected inferred that pranlukast binds to Rh5, and not basigin, providing additional evidence that pranlukast genuinely binds to Rh5 and confirms pranlukast as a hit from the screen.

During the DSF analysis we observed the addition of a high concentration (>40 μM) of pranlukast resulted in a markedly lower Tm of both Rh5 and the control protein DCLK1 (Fig. S12). These results established that pranlukast destabilised both Rh5 and DCLK1 resulting in a lower Tm. This phenomenon has been previously observed in studies by the Shoichet laboratory (Cimmperman et al., 2008; Coan et al., 2009; Doak et al., 2010). In these studies, they demonstrated pranlukast at high concentrations formed colloid aggregates that non-specifically destabilise proteins resulting in an assay artefact or a false positive. Although this is our observation with both DCLK1 and Rh5 at concentrations >40 μM of pranlukast (in which aqueous solubility is limited), at concentrations of up to 20 μM, pranlukast demonstrated a dose dependent increase in the Tm of Rh5 (Fig. 2) but did not significantly affect the Tm of the DCLK1 control protein (Fig. S12).

### 3.4 Evaluation of hit analogues

**Fig. 3:**
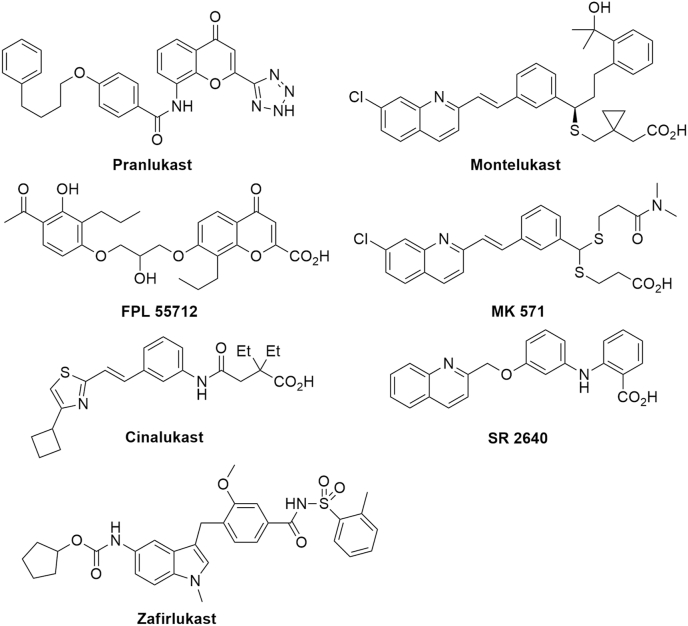
Pranlukast and related leukotriene receptor antagonists used in this study.

Demonstrating positive and negative modulation of activity through small structural changes to hit scaffold is a key step in the post screening hit confirmation process and to establish a logical structure activity relationship (SAR). We first opted to procure a set of structurally alike pranlukast analogues and evaluate them for Rh5 activity, before embarking on the synthesis of analogues. Pranlukast is one of several drugs that belong to the cysteinyl leukotriene receptor antagonist class (Fig. 3). Leukotriene is a receptor controlling leukotriene production that promotes inflammation and is an important drug target in asthma and bronchitis. Pranlukast limits the production of leukotrienes, reducing the inflammatory response in these respiratory diseases. Pranlukast has several key structural features that are common to the cysteinyl leukotriene receptor antagonist drug class, that mimic the native arachidonic acid ligands, leukotrienes. Pranlukast possesses an acidic hydrophilic group, that mimics the carboxylic acid in leukotrienes, and a rigid tether that terminates in an aromatic group that mimics the hydrophobic polyunsaturated chain of leukotriene.

We obtained several leukotriene receptor agonists that all possess similar functionality to pranlukast, to assess whether this drug class possesses generic inhibitory activity against Rh5. These drugs, shown in Fig. 3, all bind to leukotriene receptors and possess the same chemical attributes as pranlukast – an acidic hydrophilic group and an extended hydrophobic moiety. Given the commonality of these drugs to pranlukast we assessed their ability to block the Rh5 basigin interaction using the AlphaScreen assay format. All the compounds possessed binding affinities outside of the range of the assay, except for zafirlukast and FPL 55712. Zafirlukast (17 μM) and FPL 55712 (28 μM) were found to have similar activity against Rh5 compared to pranlukast (Table 1).

### 3.5 Design and synthesis of pranlukast analogues

To further confirm pranlukast is a genuine inhibitor of the Rh5 – basigin interaction, a small cohort of analogues were synthesised to help with the hit confirmation process and determine if a meaningful structure activity relationship exists. One of the defining features of pranlukast is the 2-tetrazole substitution on the chromenone. Given that the tetrazole is a known carboxylic acid isostere, we first replaced the tetrazole with an ester and carboxylic acid to assess the importance of acidic functionality in the 2-position of the chromenone. We then modified the length of the carbon chain that accommodates the terminal phenyl ring, to assess whether the length of the carbon linker was important for retaining activity.

**Fig. 4:**
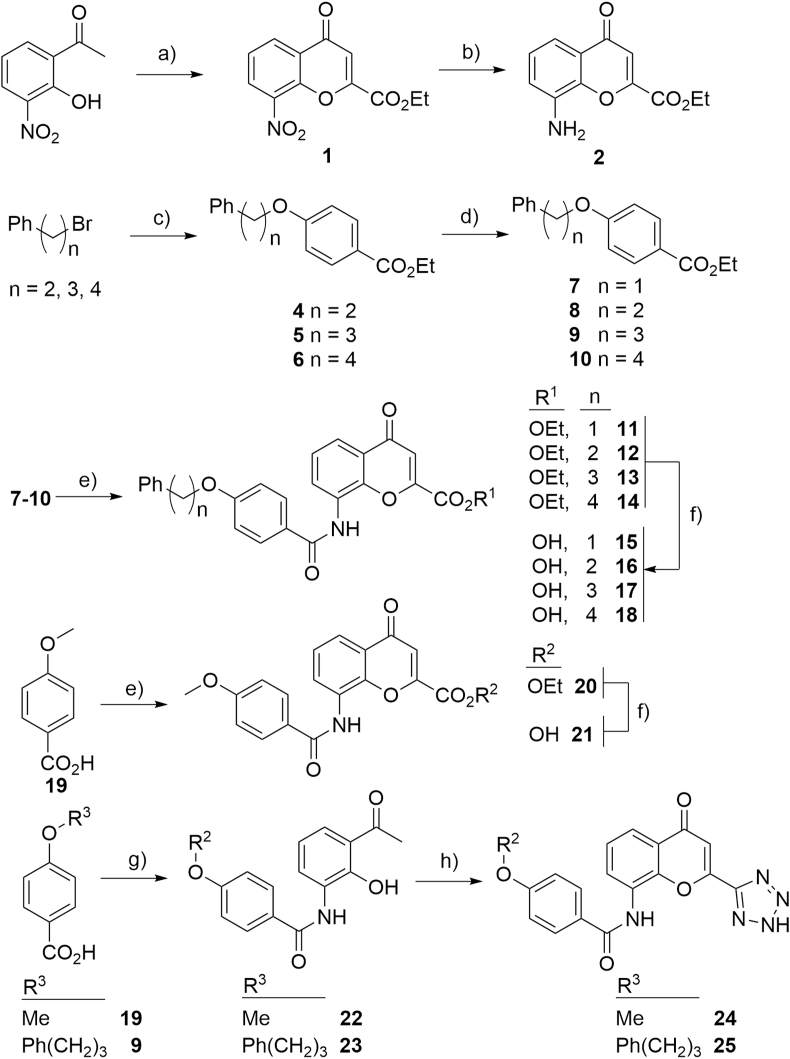
Synthesis of pranlukast analogues. *Reagents and conditions:* a) i. NaOEt, diethyl oxalate, EtOH, reflux; ii. H_2_SO_4_, 50 °C; b) SnCl_2_.2H_2_O, HCl, EtOH, 50 °C; c) ethyl 4-hydroxy benzoate, NaH, DMF, 50 °C; d) i. NaOH, THF, EtOH, H_2_O; ii. HCl; e) i. SOCl_2_, DCE, 50 °C; ii. **2**, DIPEA, DCE; f) K_2_CO_3_, H_2_O, EtOH, THF; g) i. SOCl_2_, DCE, 60 °C; ii. 1-(3-amino-2-hydroxy-phenyl) ethanone, pyridine, DCE; h) i. NaOtBu, ethyl 1H-tetrazole-5-carboxylate, DMF, 50 °C; ii. HCl, MeOH, reflux.

The synthesis of analogues to explore SAR began with the building block, ethyl 8-amino chromenone 2-carboxylate **2**, that was synthesised following a protocol previously described by Raposo et al. (1996). The synthesis was initiated by forming the enolate of 3-hydroxy-2-nitro acetophenone and reacting this with diethyl oxalate (Fig. 4). The reaction was quenched to give the 1,3-diketone intermediate. This intermediate was then heated in sulfuric acid to form the 8-nitro chromenone **1**. The nitro functionality was then reduced with stannous (II) chloride to give the 8-amino chromenone **2**. Other attempts to reduce the nitro functionality proved unsuccessful, such as hydrogenation using Pd/C that produced the tetrahydro chromanone.

The synthesis of analogues **11–18**, **20** and **21** with varying carbon chains started with alkylating ethyl 4-hydroxy benzoate (Fig. 4). The phenolate of ethyl 4-hydroxy benzoate was formed with sodium hydride and the addition of the appropriate alkyl bromide, producing **3–6** respectively. The ester functionality of **3**–**6** was then hydrolysed using sodium hydroxide to produce the carboxylic acids **7–10**. The carboxylic acids **7–10** and **19** were converted to acid chlorides using thionyl chloride, and on-reacted with the ethyl 8-amino chromenone 2-carboxylate **2** to give **11–14** and **20**. And finally, the ester group of **11**–**14** and **20** was hydrolysed to give the carboxylic acids **15–18** and **21**. It was proposed that the low yields of the hydrolysis step was due to decarboxylation of the 2-carboxylic acid group.

The synthesis of 2-tetrazole analogues **24** and **25** followed the previously described route by Geen et al. (2006). The syntheses began by reacting the acid chloride of **19** and **9** with 3-hydroxy-2-nitro acetophenone to give **22** and **23** (Fig. 4). The enolate of **22** and **23** was then formed with sodium *tert*-butoxide, and on reacted with ethyl tetrazole-5-carboxylate to form the 1,3-keto intermediates. The intermediates were heated under acidic conditions to give the desired 2-tetrazole chromenones **24** and **25**.

### 3.6 Evaluation of hit analogues to inhibit the Rh5 – basigin interaction

The synthesised pranlukast analogues were evaluated for their ability to inhibit the Rh5 basigin interaction using the AlphaScreen assay. The results of the evaluation show that the 4-carbon chain (4-C) tethered to the terminal phenyl group, as seen in pranlukast, was the optimal length for Rh5 activity (Table 1 and Fig. S10). The 3-C tether analogue **25**, bearing the 2-tetrazole, exhibited lower affinities (20 μM) compared to the 4-C tether in pranlukast (12 μM). Although the activity between compounds is not statistically significant, the trend was also observed with the carboxylic acid analogues – the 4-C tether analogue **18** (21 μM) possesses greater potency than both the 3-C tether (**17**) the 2-C tether (**16**) analogue (24 μM and 40 μM respectively). No activity was observed with the analogues that have a 1-C tether (**15**) and a methyl replacing carbon tether phenyl group (**21**), thus demonstrating that the presence of the 4-C hydrocarbon linked phenyl ring is important for Rh5 activity.

The compounds that possess the 2-ester functionality, for example compounds **12–14** were inactive, in comparison to their 2-carboxylic acid counterparts **16–18** (21–40 μM) (Table 1). This demonstrates that acidic functionality in the 2-position of the chromenone is required to block the interaction of Rh5 with basigin.

### 3.7 *P. falciparum* parasite activity of screening hits

An essential component of the evaluation cascade from a HT screen of Rh5 is to determine whether the hit or synthesised analogues of the hit reduce parasite viability by blocking merozoite invasion of the host erythrocyte. To determine this, we employed a parasite viability assay and a schizont rupture assay which can detect inhibition of merozoite RBC entry. To demonstrate of the usefulness of these assays, in a post HT screen evaluation cascade, we tested pranlukast and synthesised analogues in these assays.

Pranlukast and selected analogues were then evaluated in a *P. falciparum* parasite viability assay. For this assay, we followed the procedure described by Gamo et al. (2010), that used lactate dehydrogenase (LDH) as a readout for parasite viability. In this assay, 3D7 parasites were incubated over 72 h in the presence of increasing concentrations of compounds. The results of the *P. falciparum* viability assay described in Table 1 and Fig. S13 show that of the known leukotriene drugs evaluated, zafirlukast possesses modest activity (EC_50_ 6 μM), pranlukast is weakly active (EC_50_ 39 μM) and FPL 55712 is inactive (EC_50_ > 50 μM). Of the synthesised 2-tetrazole analogues, compound **25,** that possess a 3-C tether, was the most active (EC_50_ 7 μM), while compound **24**, that is devoid of the carbon tether, is inactive. Of the synthesised 2-carboxylate derivatives, the 3-C tether compound **17** was the most active (EC_50_ 15 μM), the 2-C (**16**) was weakly active (EC_50_ 40 μM) and the 4-C tether (**18**) derivative was inactive. For a comparison, selected compounds were incubated in the presence of *P. falciparum* D10 parasites for 72 h (Table 1, Fig. S14) and growth inhibition measured by flow cytometry. There was a good correlation between the EC_50_ values of compounds between 3D7 and D10 strains, except for compound **16** that was 5-fold more active against the D10 strain than the 3D7 strain. Overall, the activity of compounds in the *P. falciparum* viability assay have little or no correlation to the Rh5 inhibitory activity, suggesting that the modest activity observed against *P. falciparum* is off target.

**Fig. 5:**
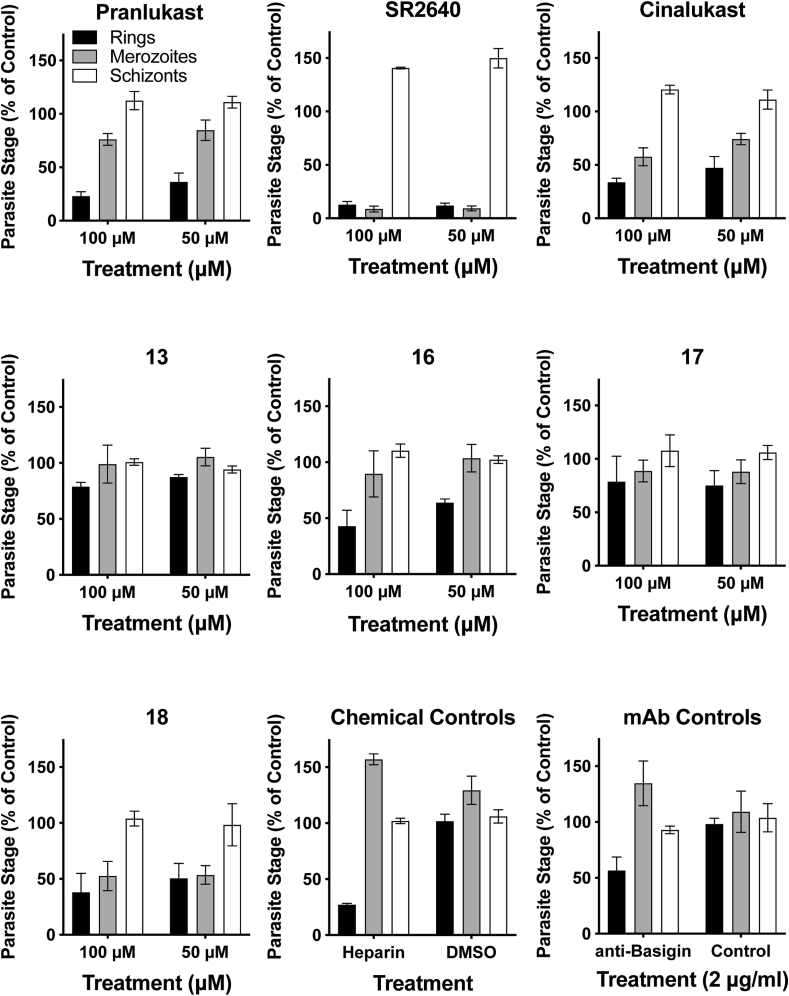
The effect of selected compounds on merozoite invasion during normal *in vitro* culture. Late schizont stage parasites (44–48 h post invasion) were allowed to rupture in the presence of compound or control antibody and parasite populations assessed 6 h later by flow cytometry. Parasite populations quantitated were newly invaded ring stages (black bar), free merozoites (grey bar) and unruptured late stage parasites (white bar). A reduction in only the ring stage parasite population as seen for the known invasion inhibitory controls heparin and anti-basigin mAb indicates specific activity against invasion. Data shown represents means for 3 independent experiments expressed as a percentage of the non-inhibitory control population. Error bars shown are SD.

To determine if the modest activity of pranlukast and analogues in the parasite viability assay was a consequence of invasion inhibition, rather than inhibition at another stage of blood stage parasite development, we performed an assay that measures the effect of compound treatment on *P. falciparum* D10 asexual stage transition between late schizont and ring stages (Wilson et al., 2013). In this assay, highly synchronised mature schizont-stage parasites were incubated with pranlukast and selected analogues at 50 and 100 μM and the transition to newly invaded ring stage parasites was quantified by flow cytometry. In parallel, the rate of schizont rupture and merozoite release was quantified to assess whether any reduction in newly invaded ring stages was due to a block affecting merozoite release or development. The invasion inhibitory controls heparin and anti-basigin mAb antibody (used at ~1 × EC_50_ (Crosnier et al., 2011)) both showed a reduction in the number of early rings stage parasites, but not free merozoites, as expected for specific merozoite invasion inhibitors (Fig. 5). Neither DMSO (at the maximal concentration used for the drug treatments of 0.1%) or a non-invasion inhibitory mAb control caused a reduction in merozoite invasion. At concentrations up to 100 μM for compounds **13** (~2 × EC_50_) and **17** (~5 × EC_50_) there was minimal inhibition of merozoite invasion, schizont rupture and no reduction in free merozoite numbers, indicating that these compounds have no activity against merozoite development or invasion. Compounds SR2640 (100 μM: ~16 × EC_50_), cinalukast (100 μM: ~3 × EC_50_) and **18** (100 μM: ~2 × EC_50_) all showed a reduction in numbers of merozoites that had invaded and formed early ring stages, but all also showed similarly reduced numbers of free merozoites indicating that these drugs inhibited merozoite development or schizont rupture rather than merozoite invasion. In the case of SR2640 at the concentrations tested, there was near complete loss of the free merozoite population associated with a >40% increase in unruptured schizonts, indicating that this drug has significant activity against schizont development. For pranlukast (100 μM: ~4 × EC_50_) and compound **16** (100 μM: ~16 × EC_50_), there was a substantial reduction in the numbers of merozoites that had invaded and formed early ring stages. At the lowest concentration tested (50 μM), there was minimal loss in free merozoite numbers for both pranlukast and **16**, indicating that invasion inhibition was predominantly against the merozoite rather than the developing schizont at this concentration. In summary of the schizont rupture assay results, pranlukast and analogues appear to inhibit schizont rupture or merozoite development and not merozoite invasion of the RBC, suggesting the activity observed is not related to the inhibition of Rh5. The data obtained here, demonstrates the appropriateness of this assay in the analysis of hit compounds originating from future HT screens against Rh5.

## 4 Discussion

Rh5 is essential for the ability of the *P. falciparum* parasite to invade the erythrocyte and for its survival (Crosnier et al., 2011). Previous studies have shown that antibodies to Rh5 (Chiu et al., 2014; Douglas et al., 2014) or basigin (Reddy et al., 2014) block the entry of the malaria parasite into the erythrocyte, and therefore Rh5 is a promising vaccine target and potentially a small molecule therapeutic target. To identify small molecules that block binding of Rh5 to basigin, we developed a biochemical assay using AlphaScreen technology in 384-well format that has the capacity to be used in a high throughput approach to screen large collections of small molecules. To determine the suitability of the assay for this role, we conducted a pilot screen using a small set of known drugs and a collection of compounds with antimalarial properties. Statistical analysis of this screen demonstrated high reproducibility with a Z’ prime value of >0.8 and high signal-to-noise values across plates (Figs. S4 and S5) suggesting the assay format is suitable for screening larger libraries. The screen was conducted in a 384-well format, and although we did not attempt to miniaturise the assay for a 1536-well format to reduce assay costs and increase throughput, the statistics of the pilot screen imply the assay would be adaptable to a 1536-well format.

We used two standard deviations of the mean to increase the hit rate of the primary screen, as HT screens on PPIs generally have a lower hit rate. Applying this measure, the hit cut-off rate was approximately 50% inhibition of Rh5 for both libraries screened and gave a total of 231 hits (Fig. S6). Three standard deviations of the mean is normally applied to primary screens to determine the number of hits, and this measure could be applied to screening larger compound libraries. Ultimately, the confirmation and counterscreen applied eliminated the high number of hits obtained from the primary pilot screen of the known drug and MMV Malaria Box libraries.

To validate the hits from the primary pilot screen, we conducted confirmation and counter screens firstly in single point and then in a 11 pt dose response format. The TruHits assay format was used for the conuterscreen and eliminated a large number of false positives that interfere with the AlphaScreen signal (Figs. S8 and S9). The post screening measures applied to the screen of the MMV Malaria Box identified no compounds that inhibit the Rh5-basigin interaction, whereas the screen of the known drug libraries identified pranlukast as a low micromolar inhibitor of the Rh5 – basigin interaction (Fig. 1).

DSF is one biophysical technique that was utilised as a post screening measure to aid in the confirmation of hits from a screen against Rh5. Surface plasmon resonance (SPR) is another biophysical assay that could also employed to verify binding of compounds to Rh5, however in the instance of pranlukast, this technique could not be used because of the limited solubility of pranlukast in the SPR running buffer. Therefore, DSF was used to confirm that pranlukast at concentrations near to the AlphaScreen IC_50_ value (Table 1) binds to and stabilises Rh5 (Fig. 2). Pranlukast has been previously identified to form colloidal aggregates at high micromolar concentrations in certain biochemical assay conditions (Doak et al., 2010). Shoichet et al. found that these aggregates bind to, sequester, and inhibit the function of proteins non-specifically in the absence of a detergent. Shoichet et al. also showed that ligand formed colloidal aggregates, may bind to a protein and induce a structural conformational change, resulting in a decrease in Tm as measured by thermal shift analysis (Coan et al., 2009). Consistent with the findings of Shoichet et al., we also observed that at concentrations of >40 μM, pranlukast destabilised both Rh5 and DCLK1 (Fig. S12), which raises the possibility that pranlukast is a false positive screening hit due to its potential aggregating properties. However, there are several reasons why pranlukast may not be a false-positive in this instance. Firstly, the non-ionic surfactant, Tween 20, was used in the AlphaScreen assay to identify pranlukast. The addition of Tween 20 was shown by Shoichet et al. to suppress aggregate formation (Coan et al., 2009). In addition, casein was also added to the AlphaScreen assay as a carrier protein to further sequester and nullify the effects of colloidal aggregates. Secondly, analogues similar in structure to pranlukast, such as **13**, **14** and **15**, are inactive in the AlphaScreen assay, and finally, at concentrations near to the IC_50_ of pranlukast in the AlphaScreen assay, pranlukast stabilised Rh5 (Fig. 2) but not the DCLK1 control protein (Fig. S11) using DSF. Collectively, these data suggest that pranlukast is binding to Rh5 and therefore it is likely pranlukast is a genuine screening hit.

Procurement and synthesis of hit analogues also assists in the hit confirmation process, by demonstrating that distinct changes to the structure of the hit molecule results in meaningful positive or negative changes in activity. This is particularly pertinent with hits from a screen of a PPI, whereby relatively large structural changes result in subtle changes to activity. We used this approach to help dissect whether changes to the pranlukast scaffold modulate Rh5 activity to give logical SAR. Pranlukast is an orally bioavailable leukotriene antagonist that is used for treatment of bronchial dilations. All marketed leukotriene antagonists mimic the natural substrate leukotriene, a long chain arachidonic acid. The leukotriene drugs have two defining features, a carboxylic acid (or acidic moiety) and an extended hydrophobic domain. Several other commercially available leukotriene drugs share these two features. We postulated that other leukotriene drugs contain a similar pharmacophore to pranlukast, and therefore may interact with Rh5 in the same manner as pranlukast. We procured six leukotriene antagonists, zafirlukast, montelukast, cinalukast, SR 2640, FPL 55712 and MK 571 (Fig. 3), and screened these using the Rh5 AlphaScreen assay. Zafirlukast and FPL 55712 were found to both possess inhibitory activity, or albeit with lower activity compared to pranlukast (Table 1, Fig. S10). Zafirlukast, like pranlukast, has a large hydrophobic region and an acyl sulfonamide imitating a carboxylic acid. While FPL 55712 has greater similarity, sharing the same 2-substituted chromenone as pranlukast. Montelukast, cinalukast and MK 571 did not show Rh5 inhibitory activity at the highest concentration tested (50 μM). The small selection of synthesised pranlukast analogues suggested that the acidic functionality is essential for Rh5 activity, and thus masking the acidic functionality with an ester (compounds **16** and **17**) completely ablates activity.

The small selection of synthesised pranlukast analogues (Fig. 4) also demonstrated that alterations to the length of the carbon chain terminating in a phenyl group was important for activity (Table 1, Fig. S10). The activity of analogues **15–18** and **25**, show that shortening the tether, or removing this functionality entirely (compound **21** and **24**), correlates with a reduced Rh5 inhibitory activity. In the absence of a structural model of these analogues bound to Rh5, it is not known why shortening the tether is detrimental to Rh5 activity. It is unlikely this is due to a small reduction in lipophilicity, but probable that the terminating phenyl group is partaking in a key interaction that enables the binding of pranlukast to Rh5. There is a possibility that the α,β-unsaturated moiety of the chromenone is acting as a Michael acceptor. This moiety could react non-specifically with a non-disulfide linked Cys on either Rh5 or basigin (Cys329 or Cys137 respectively), forming an irreversible covalent bond. However, the structure activity relationship observed with the small cohort of compounds synthesised and evaluated here, would suggest pranlukast does not react with a non-disulfide linked Cys. For example, compounds **13** and **14** possess functionality capable of acting as a Michael acceptor, but exhibit no activity, therefore demonstrating pranlukast is not acting through this mechanism.

In the next phase of the post screening evaluation cascade, we employed two assays to determine if the hits from the Rh5 screen and synthesised analogues reduce parasite viability by blocking merozoite invasion of the host erythrocyte. The first assay utilised a parasite viability assay and the second a schizont rupture assay capable of detecting inhibition of merozoite entry into the host erythrocyte. To demonstrate the utility of these assays in a post HT evaluation cascade for Rh5 we used pranlukast as a model. Pranlukast and selection of analogues were first evaluated for their effectiveness in 72 h growth inhibition assays using 3D7 and D10 strains of *P. falciparum* (Table 1, Figs. S13 and S14). The modest parasite growth inhibitory activity observed did not correlate with the Rh5 inhibition values, indicating the parasite growth inhibitory activity was not a result of Rh5 inhibition. It is unlikely the compounds physicochemical properties (PSA, cLogP) were a factor in the differential parasite activity observed, because these are relatively similar, and it is generally expected for an analogue series to have robust correlation between cellular and biochemical activity. Furthermore, the activity observed in the *P. falciparum* schizont to ring development assay (Fig. 5) shows the majority of pranlukast analogues either have minimal activity against merozoite invasion (compounds **13**, and **17**) or non-specifically affect merozoite development and/or release from the late stage schizont (SR2640, cinalukast and **18**), demonstrating these compounds are not impacting parasite growth by blocking erythrocyte invasion. Both pranlukast and compound **16** showed evidence of direct inhibition of invasion, with the loss in invasion substantially greater than the minimal reduction in merozoite development also observable for these compounds. However, for both pranlukast and compound **16** invasion inhibition was incomplete at concentrations ~2 × EC_50_ and ~8 × EC_50_ at 72 h for D10 parasites respectively, indicating loss of merozoite invasion is only a partial contributor for the growth inhibitory activity of these compounds. We conclude that the biochemical inhibition of Rh5 by pranlukast and analogues is too weak for merozoite invasion inhibition to be a major contributor to the growth inhibitory activity of these compounds, even at the high concentrations tested.

Our data suggests that pranlukast is an inhibitor of Rh5, although the limited solubility of pranlukast in certain conditions, prevented its further evaluation in techniques such as SPR, and therefore without this supporting data we cannot conclusively state that pranlukast is a genuine Rh5 inhibitor. Furthermore, there was no robust correlation between biochemical inhibition of Rh5 and parasite activity, bringing into question the developability of pranlukast as an RBC invasion inhibitor. Nevertheless, the Rh5 biochemical assay developed here demonstrates its future utility in screening large compound libraires to discover Rh5 inhibitors that have suitable physical and structural attributes for further optimisation.

An additional consideration for future screening, are the client proteins necessary for the essential function of Rh5. Rh5 forms a complex with both Ripr and CyRPA (Reddy et al., 2015) that allows it to be inserted into the host membrane. It is known that these proteins form a trimeric complex with Rh5 binding only to CyRPA (Wong et al., 2018). It is also known that Rh5 appears to oscillate between two major conformations and that the Rh5-CyRPA-Ripr complex binds significantly better to basigin (Wong et al., 2018). Therefore, it is likely that it would be advantageous to screen libraries of small molecules against entire Rh5-Ripr-CyRPA complex and basigin, compared to our approach here of Rh5 alone with basigin.

## 5 Conclusions

Rh5 has been implicated as an important and promising vaccine target against *P. falciparum* (Drew and Beeson, 2015). Studies using antibodies to both Rh5 have shown that entry of the merozoite into erythrocytes is blocked (Chiu et al., 2014; Douglas et al., 2014; Healer et al., 2019; Tran et al., 2014). Given this promising set of results using antibodies against Rh5, a small molecule inhibitor of Rh5 may be equally effective in preventing entry of the merozoite into the red blood cell. Here, we developed an assay with the capability to screen large compound libraries to identify small molecule inhibitors of the Rh5 - basigin interaction. To demonstrate the utility and the robustness of the assay, we conducted a pilot screen using a small library of compounds and identified pranlukast as a hit. We suggest several assays and approaches that could be used in the post screening evaluation cascade of an Rh5 screen. We used pranlukast as a model compound for this purpose and showed that although pranlukast was able to stabilise Rh5 by DSF analysis, the Rh5 inhibitory activity of pranlukast was too weak to effectively block merozoite entry. In summary of this data, it is uncertain whether pranlukast represents a suitable starting point for further medicinal chemistry program optimisation. Therefore, screening large compound libraires using the Rh5 biochemical assay developed here may uncover starting points with improved attributes for development as an antimalarial that blocks merozoite entry into the host RBC.

It is not known whether a small molecule inhibitor of merozoite invasion will be appropriate in an *in vivo* or in a clinical setting as a prophylactic or a drug treatment, but a number of groups around the world are pursuing the strategy of developing invasion inhibitory drugs to treat malaria (reviewed in (Burns et al., 2019)). Furthermore, it is not certain if a PPI is an appropriate target for an antimalarial therapeutic because of the significant cost in producing a large, complex, small molecule required to inhibit a short-term PPI interaction, and therefore it may prove difficult to develop such an inhibitor against Rh5 that meets the WHO guidelines of less than $1 per treatment. Nevertheless, discovery of a Rh5 inhibitor would serve as a valuable tool for further studying *P. falciparum* erythrocyte invasion specifically mediated by Rh5.

## Declaration of competing interest

We wish to confirm that there are no known conflicts of interest associated with this publication and there has been no significant financial support for this work that could have influenced its outcome.

We confirm that the manuscript has been read and approved by all named authors and that there are no other persons who satisfied the criteria for authorship but are not listed. We further confirm that the order of authors listed in the manuscript has been approved by all of us.

We confirm that we have given due consideration to the protection of intellectual property associated with this work and that there are no impediments to publication, including the timing of publication, with respect to intellectual property. In so doing we confirm that we have followed the regulations of our institutions concerning intellectual property.

We understand that the Corresponding Author is the sole contact for the Editorial process (including Editorial Manager and direct communications with the office). He/she is responsible for communicating with the other authors about progress, submissions of revisions and final approval of proofs. We confirm that we have provided a current, correct email address which is accessible by the Corresponding Author and which has been configured to accept email from sleebs@wehi.edu.au.

## Abbreviations

ACT: artemisinin combination therapy
AlphaScreen: amplified luminescent proximity homogeneous assay screen
BCL-2: B cell lymphoma 2
BCL-XL: B-cell lymphoma extra large
CyRPA: cysteine-rich protective antigen
DCLK1: doublecortin like kinase 1
DSF: differential scanning fluorimetry
HT: high throughput
LDH: lactate dehydrogenase
mAb: monoclonal antibody
MMV: Medicines for Malaria Venture
PEMS: parasitophorous vacuole enclosed membrane structures
PPI: protein-protein interaction
PSA: polar surface area
RBC: red blood cell
Rh5: reticulocyte binding-like homologue 5
Ripr: Rh5-interacting protein
SAR: structure and activity relationship
