# Supplementary material for: Development and application of a high-throughput screening assay for identification of small molecule inhibitors of the P. falciparum reticulocyte binding-like homologue 5 protein
Source: Int J Parasitol Drugs Drug Resist. 2020 Oct 29;14:188–200. doi: 10.1016/j.ijpddr.2020.10.008 (PMC7645381; doi:10.1016/j.ijpddr.2020.10.008)

***SUPPLEMENTARY INFORMATION FOR***

**Development and application of a high-throughput screening assay for identification of small molecule inhibitors of the *P. falciparum* reticulocyte binding-like homologue 5 protein**

Brad E. Sleebs, Kate E. Jarman, Sonja Frolich, Wilson Wong, Julie Healer, Weiwen Dai, Isabelle S. Lucet, Danny W. Wilson, Alan F. Cowman

**Index**

Page

2 Figure S1. Schematic of the AlphaScreen assay format

3 Figure S2. AlphaScreen development data

4 Figure S3. The effect of DMSO concentration on the AlphaScreen signal.

5 Figure S4. Z’ analysis across plates for primary screen.

6 Figure S5. Signal-to-noise ratios across plates for primary screen.

7 Figure S6. Frequency of distribution of hits from the primary screen.

8 Figure S7. Frequency of distribution of hits from the confirmation screen.

9 Figure S8. Scatter plot of correlation between confirmation and counterscreen (single pt).

10 Figure S9. Scatter plot of correlation between confirmation and counterscreen (11 pt).

11 Figure S10. Rh5 AlphaScreen dose response curves of pranlukast analogues.

12 Figure S11. DSF analysis of DCLK1 at low concentrations of pranlukast.

13 Figure S12. DSF analysis of Rh5 and DCLK1 at high concentrations of pranlukast.

14 Figure S13. *P. falciparum* 3D7 parasite viability dose response data.

15 Figure S14. *P. falciparum* D10 parasite growth inhibition dose response data.

15-20 Chemistry experimental.

21 References.

22-32 ^1^H NMR and LCMS of biologically evaluated compounds.

**Tables provided as a separate Excel spreadsheets.**

Table S1. Data for the primary screen, confirmation and counterscreen (at 20 μM) of the known drug library and the MMV Malaria Box against *P. falciparum* Rh5.

Table S2. IC_50_ data for the confirmation and counterscreen (11 pt titrations) of the 45 hits from the screen of the known drug library and the MMV Malaria Box against *P. falciparum* Rh5.


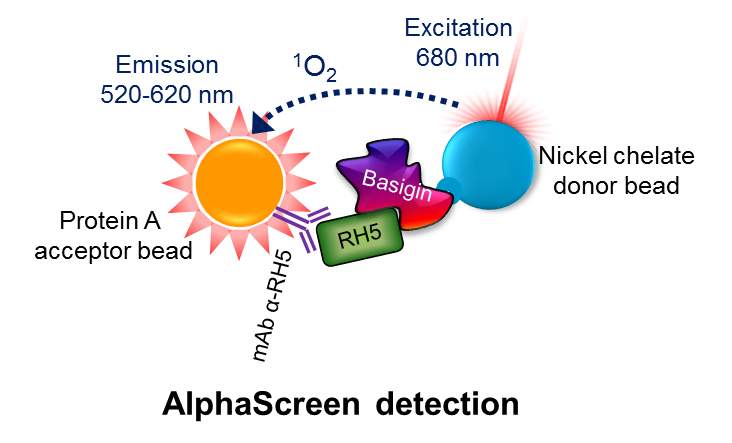


**Figure S1.** Schematic of the AlphaScreen assay format used to screen known drug libraries and the MMV Malaria Box to identify inhibitors of the Rh5-basigin interaction.


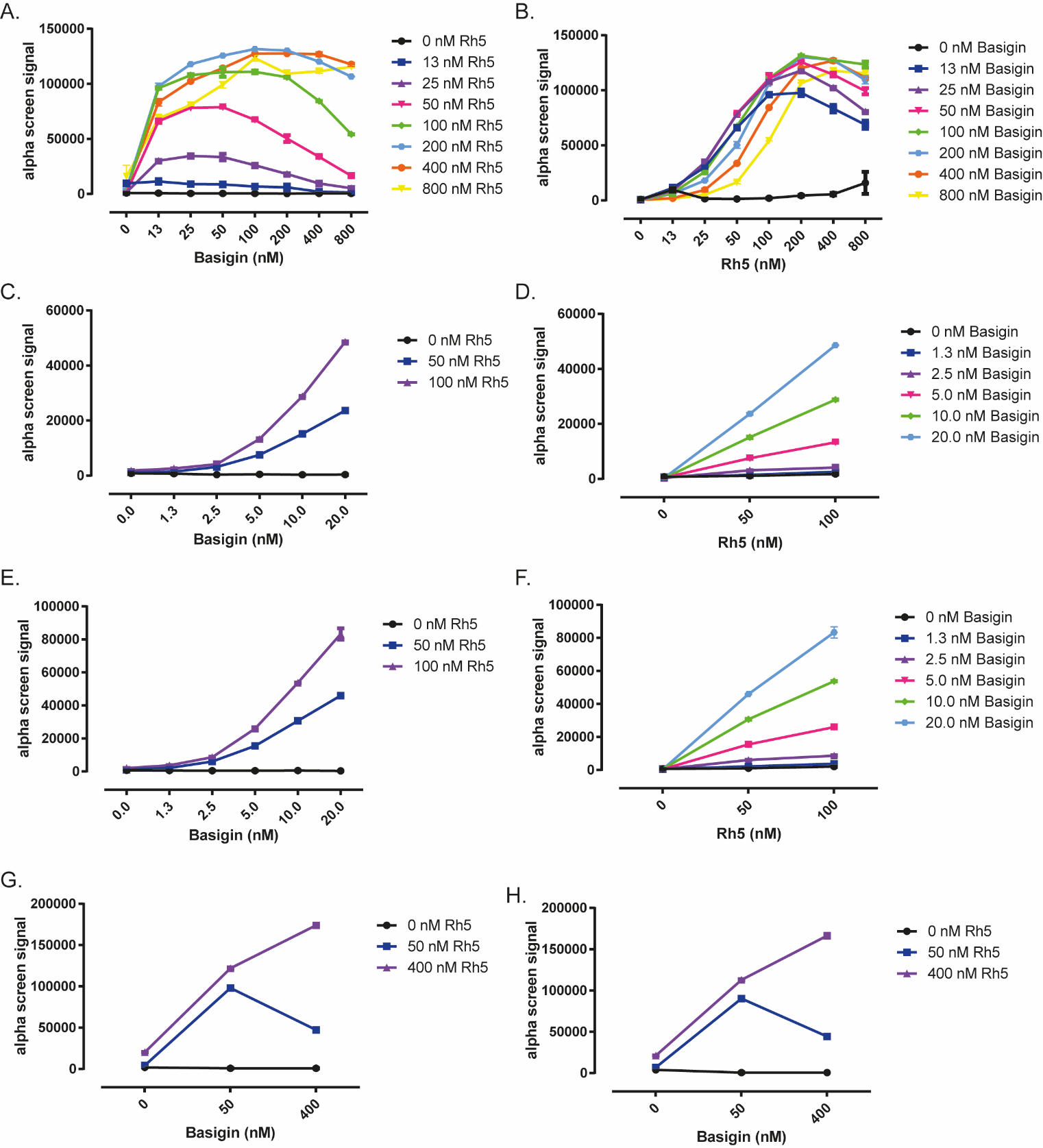


**Figure S2.** The effect of increasing concentrations of Rh5, basigin and Rh5 mAb and incubation time on the AlphaScreen signal. Data represents an average of three or more experiments. Error bars indicate SD. A) and B). Standard curves of Rh5 and basigin using 2 μg/mL Rh5 mAb. C) and D) Standard curves of Rh5 and basigin, at lower concentrations of basigin using 2 μg/mL Rh5 mAb. E) and F) Standard curves of Rh5 and basigin, at lower concentrations of basigin using 1 μg/mL Rh5 mAb. Standard curves of Rh5 and basigin using 1 μg/mL of Rh5 mAb with either a G) 1 h or H) 5 h AlphaScreen bead incubation.

**

**

**Figure S3.** The effect of increasing DMSO concentration on the AlphaScreen signal. Conditions: 1 μg/mL of Rh5 mAb, 20 nM basigin, RH5 50 nM with 1 h AlphaScreen incubation time. Data represents an average of two experiments. Error bars indicate SD.

**A.**


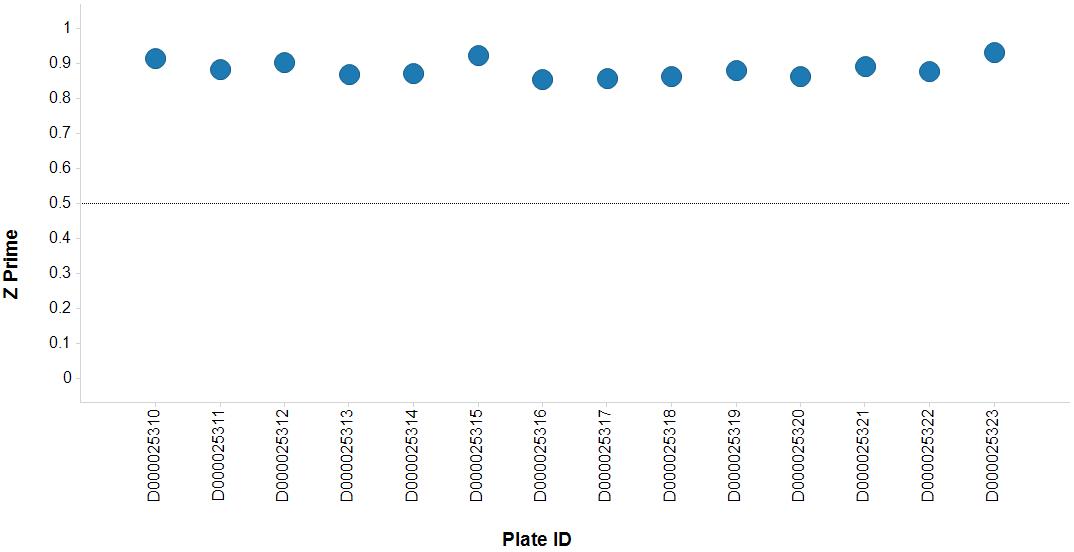


**B.**


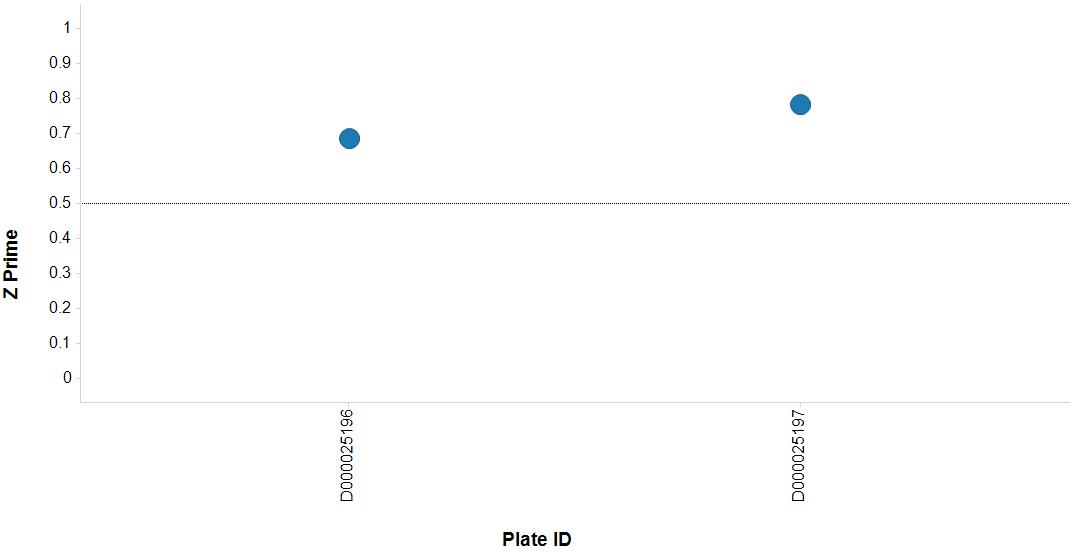


**Figure S4.** Z’ analysis across plates for primary screen of the known drug library (A) and the MMV Malaria Box (B) against *P. falciparum* Rh5.

**A.**


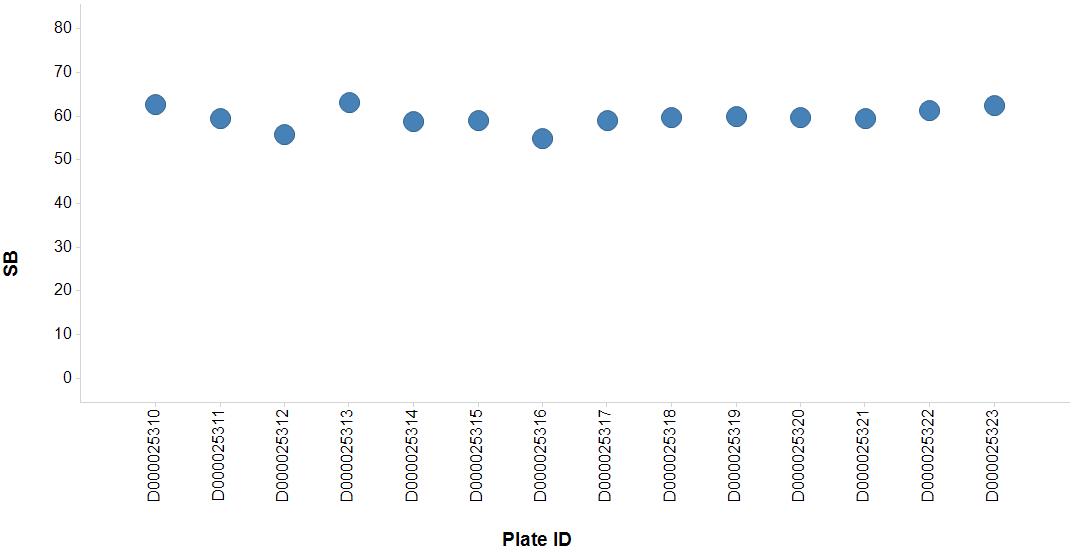


**B.**


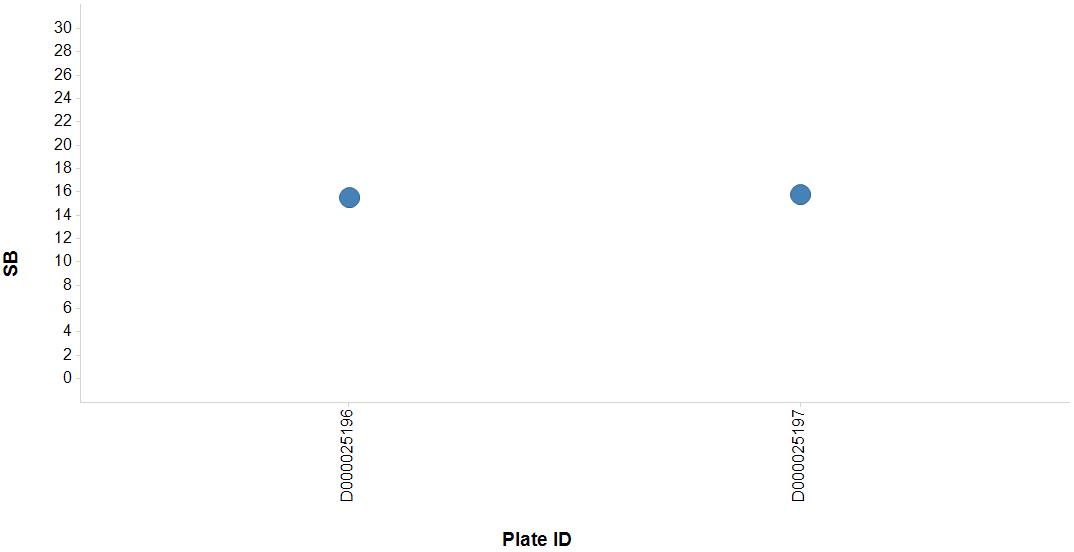


**Figure S5.** Signal-to-background (SB) ratios across plates for primary screen of the known drug library (A) and the MMV Malaria Box (B) against *P. falciparum* Rh5.


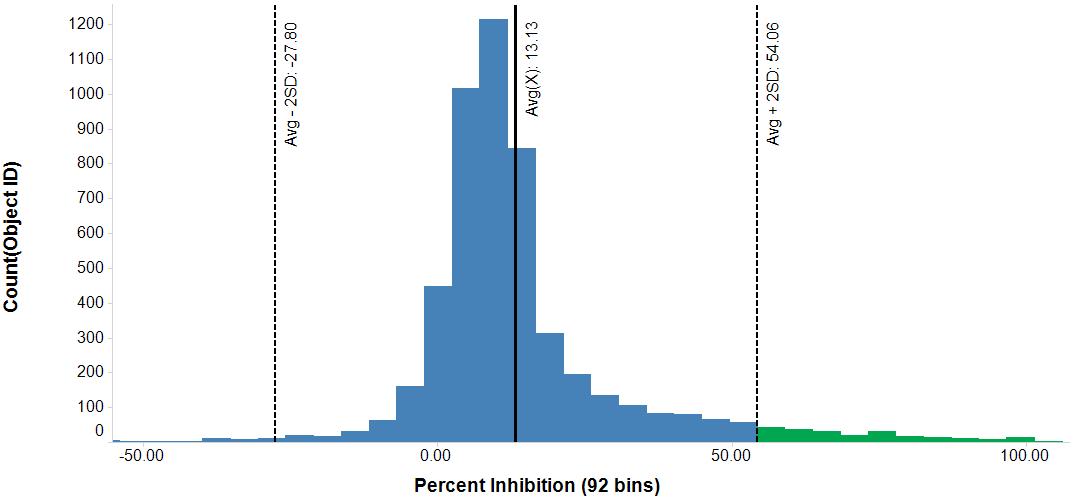


**Figure S6.** Frequency of distribution of hits from the primary screen of both the known drug library and the MMV Malaria Box against *P. falciparum* Rh5. Compounds with greater than two standard deviations of the mean or greater than 54% at 20 μM were considered primary hits.


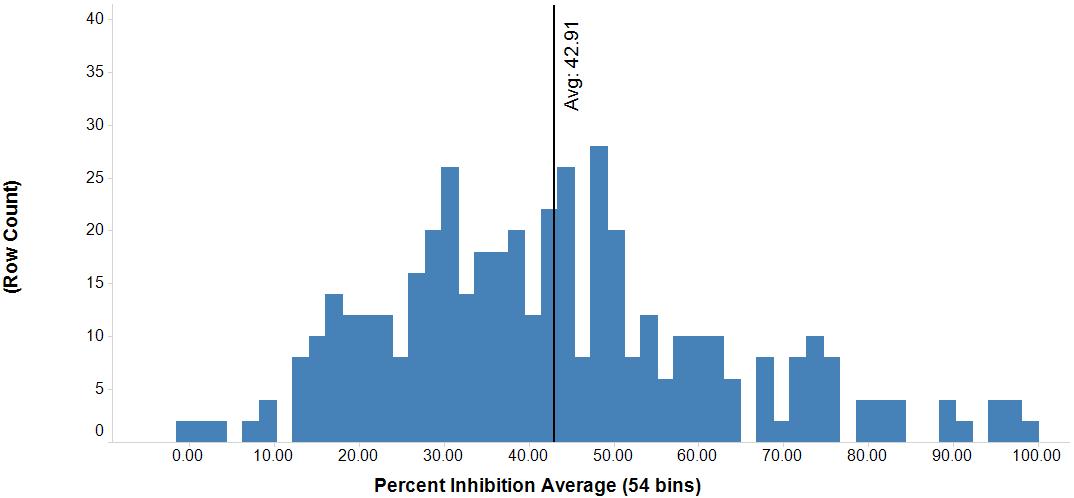


**Figure S7.** Frequency of distribution of the 231 hits from the confirmation screen of both the known drug library and the MMV Malaria Box against *P. falciparum* Rh5. Compounds that displayed greater than 50% inhibition were considered confirmed and taken forward to 11 pt dose response titrations.


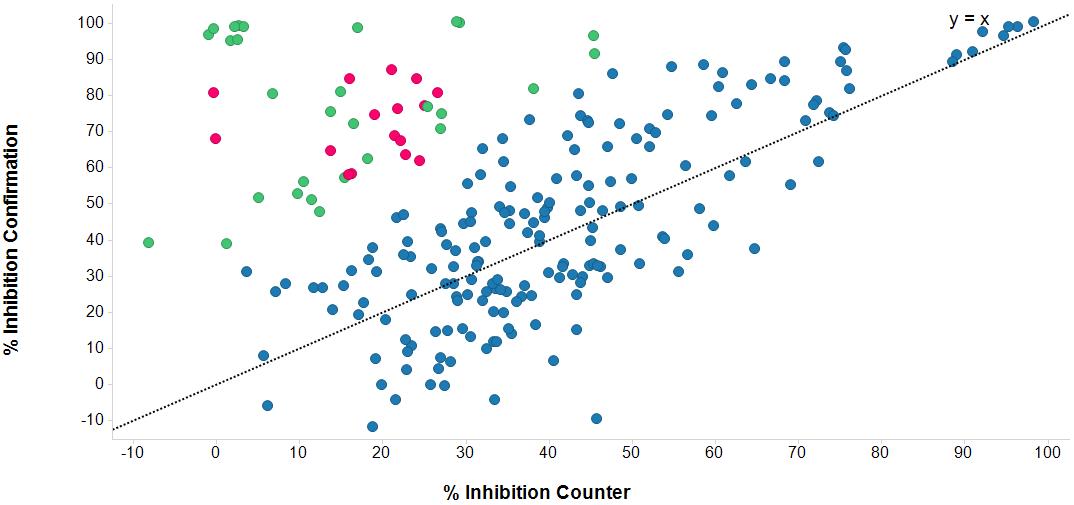


**Figure S8.** Scatter plot of correlation between confirmation and TruHits counterscreen single pt (20 μM) assays. Hits from both the known drug library and the MMV Malaria Box are indicated. The green (known drug) and red dots (Malaria Box) represent the 45 hits taken to 11 pt titrations.


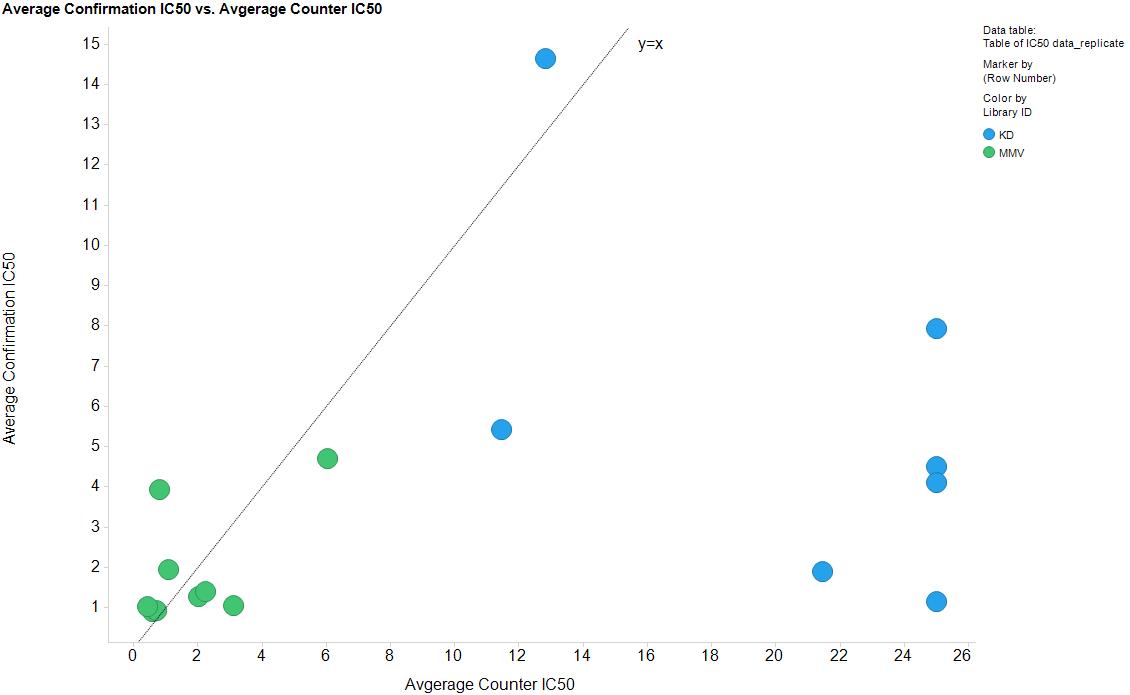


**Figure S9.** Scatter plot of correlation between confirmation and TruHits counterscreen 11 pt titration assays. Hits from both the known drug (KD) library and the MMV Malaria Box are indicated. The box represents the 5 highest ranked hits.

**
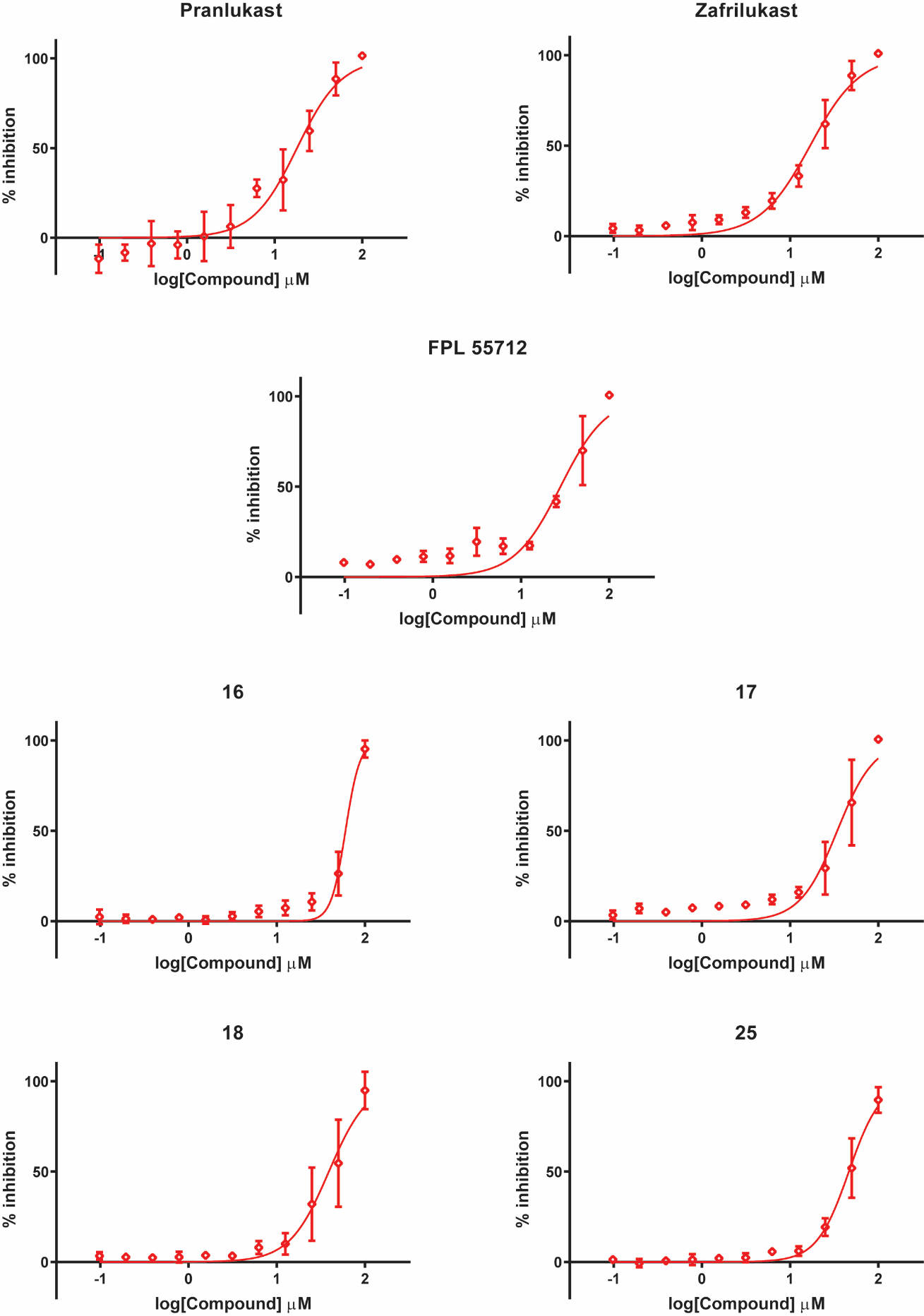
**

**Figure S10.** *P. falciparum* Rh5 AlphaScreen IC_50_ dose response curves of active compounds. An 11-point dilution series of each compound in duplicate was incubated at 20°C with Rh5. Data represents means for 3 independent experiments. Error bars are SD.


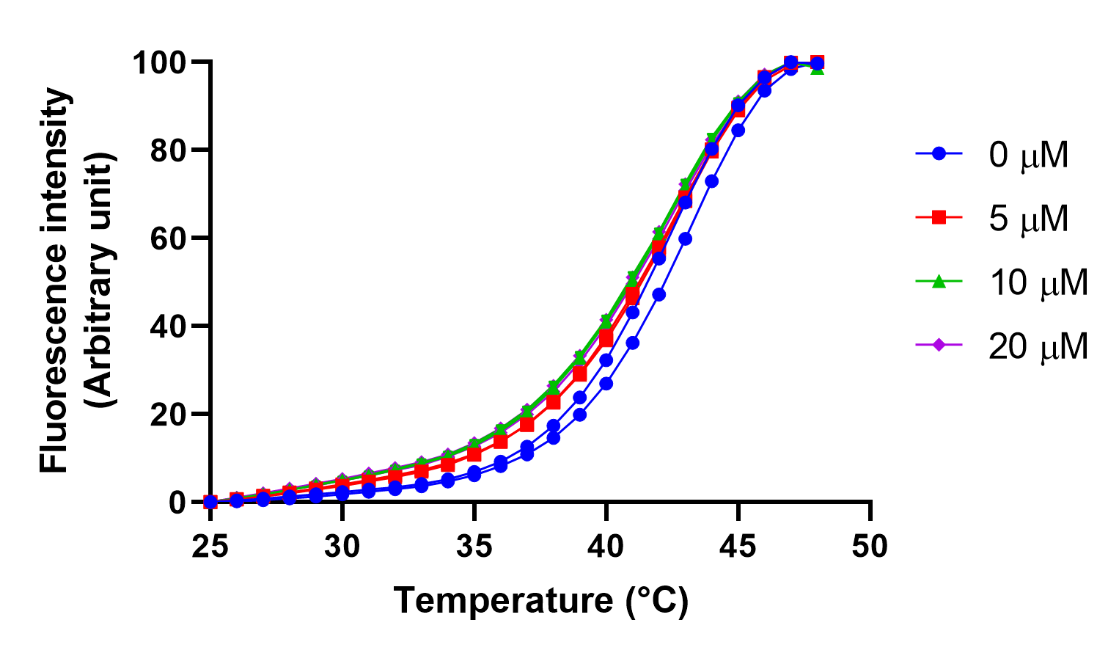


**Fig S11.** Differential scanning fluorimetry analysis of the control protein DCLK1 using pranlukast at concentrations of 0 - 20 μM showing no effect on the stabilisation of DCLK1. Data shown represents two independent experiments.


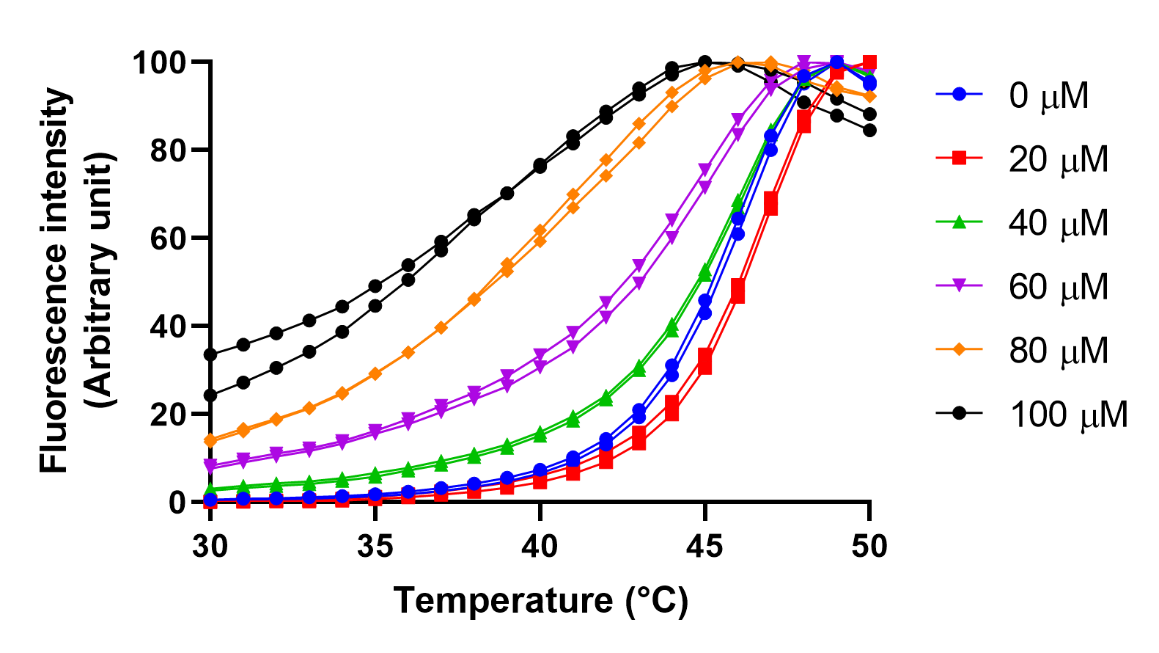


**Figure S12A.** Differential scanning fluorimetry analysis of Rh5 using pranlukast at concentrations of 0 to 100 μM showing stabilisation of the Rh5 protein at 20 μM, but destablisation of Rh5 at higher concentrations of pranlukast. Data shown represents two independent experiments.


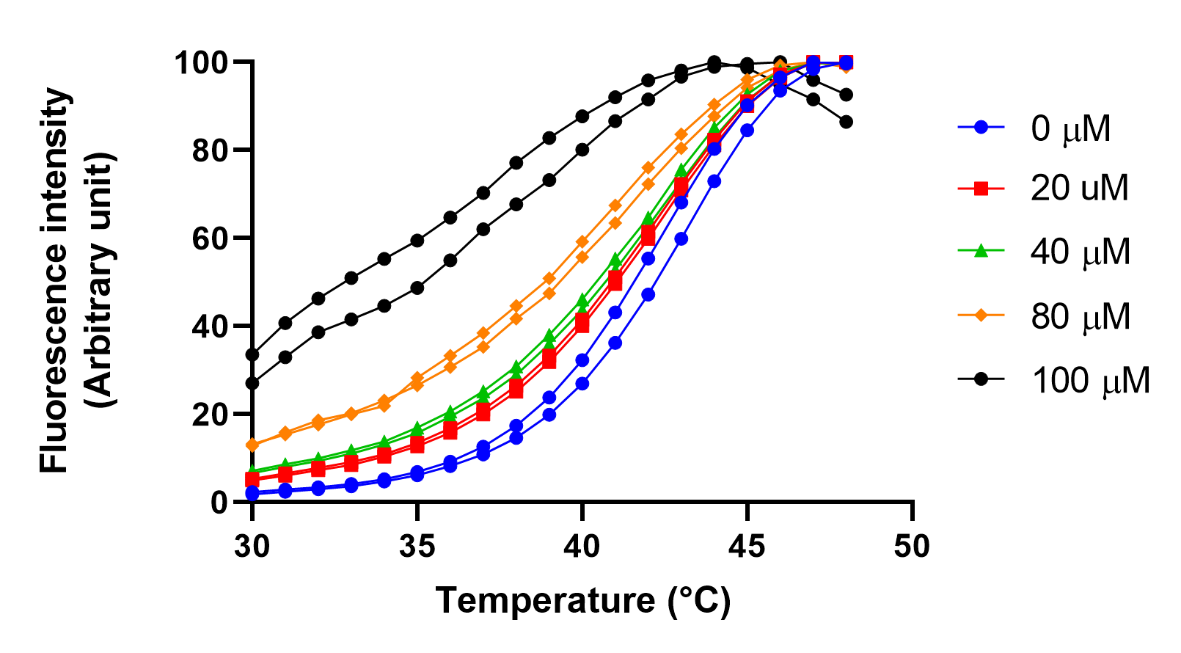


**Figure S12B.** Differential scanning fluorimetry analysis of the control protein DCLK1 using pranlukast at concentrations of 0 -100 μM showing destablisation of DCLK1 at higher concentrations. Data shown represents two independent experiments.

**

**

**Figure S13.** Dose response curves of selected compounds against *P. falciparum* 3D7. EC_50_ data represents means of three experiments measuring LDH activity of *P. falciparum* 3D7 parasites following exposure to compounds in a 10-point dilution series for 72 h. For 3D7, chloroquine EC_50_ 18 nM; Brefeldin A EC_50_ 3.5 μM. Error bars are SD.

**
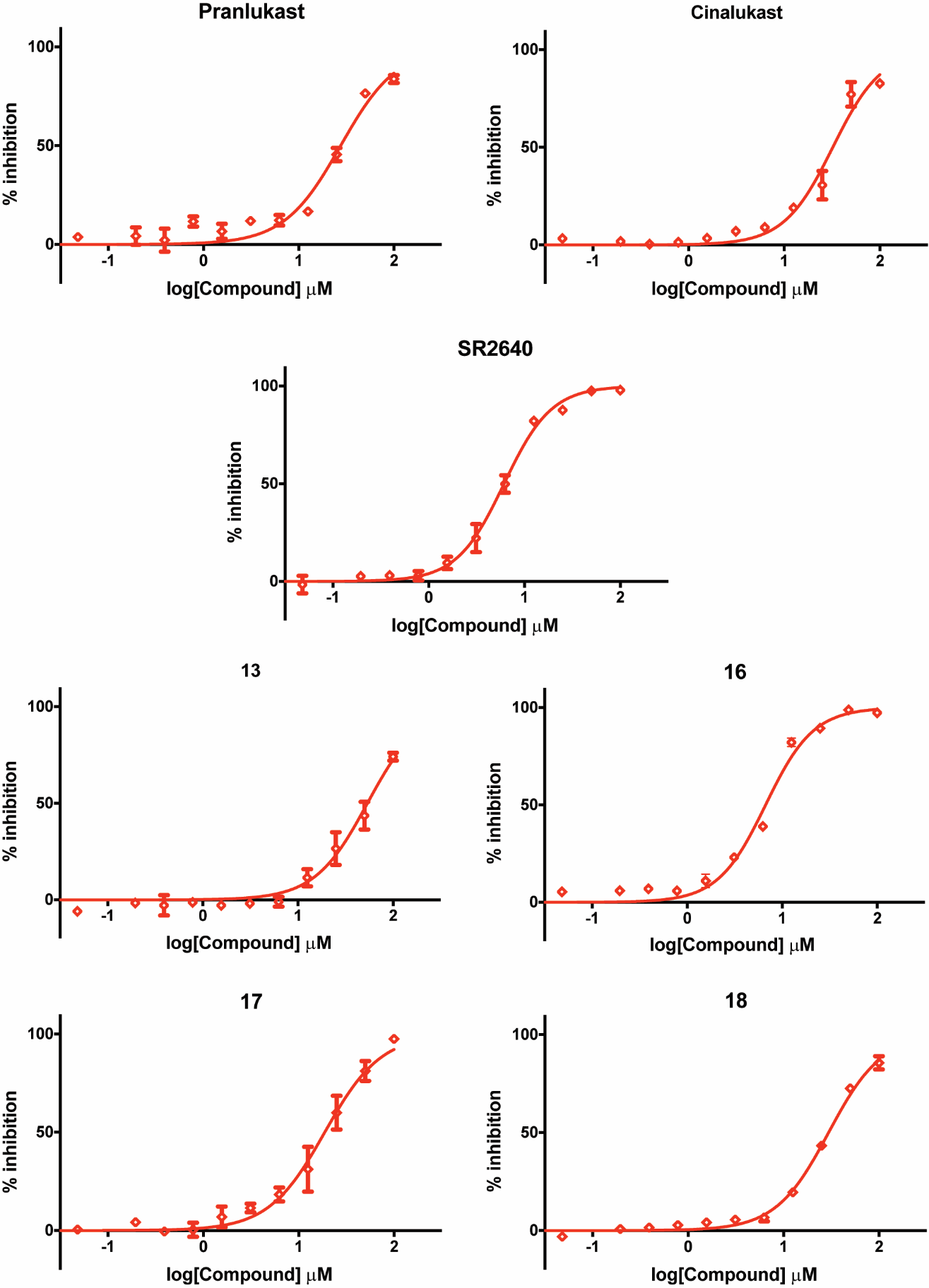
**

**Figure S14.** Dose responses of selected compounds against *P. falciparum* D10 in a growth inhibition assay. Data represents means of three experiments measuring growth inhibition by flow cytometry following exposure to compounds in a 12-point dilution series for 72 h. Error bars are SD.

**Chemistry Experimental**

Analytical thin-layer chromatography was performed on Merck silica gel 60F^254^ aluminum-backed plates and were visualized by fluorescence quenching under UV light or by KMnO_4_ staining. Flash chromatography was performed with silica gel 60 (particle size 0.040-0.063 µm). NMR spectra were recorded on a Bruker Avance DRX 300 or a Varian 600 MHz at 298K unless specified with the solvents indicated. Chemical shifts are reported in ppm on the δ scale and referenced to the appropriate solvent peak. d_6_-DMSO and CDCl_3_ contain H_2_O. All final compounds were analyzed using a Waters ZQ 3100 using a 2996 Diode Array Detector. LCMS conditions used to assess purity of compounds were as follows, column: XBridge TM C18 5 µm 4.6 x 100 mm, injection volume 10 µL, gradient: 10-100% B over 10 min (solvent A: water 0.1% formic acid; solvent B: AcCN 0.1% formic acid), flow rate: 1.5 mL/min, detection: 254 nm.

The following compounds were purchased commercially and used without further purification, 4-(benzyloxy)benzoic acid **7**, 4-methoxybenzoic acid **19**, 2-hydroxy-3-nitroacetophenone and 1-(3-amino-2-hydroxyphenyl)ethanone.

**Ethyl 8-nitro-4-oxo-4H-chromene-2-carboxylate** **(1).** Sodium (1.14 g, 48 mmol) was dissolved in EtOH (50 mL). Diethyl oxalate (3.44 mL, 26 mmol) and 3-hydroxy-2-nitro acetophenone (2.0 g, 11.0 mmol) were then added. The solution was heated to reflux for 20 min. The reaction was then quenched with 2N hydrochloric acid (100 mL). The solution was extracted with EtOAc (2 x 20 mL) and the organic layer was washed with water dried (MgSO_4_) and concentrated *in vacuo*. The solid was subjected to silica chromatography gradient eluting with 100% DCM to 5% MeOH/DCM to obtain a yellow solid. The solid was then dissolved in concentrated sulfuric acid (10 mL) and heated to 50^o^C for 10 min. Ice water (100 mL) was added and the solution extracted with EtOAc (2 x 40 mL). The organic layer was washed with water (100 mL) and the organic layer was dried (MgSO_4_) and concentrated *in vacuo*. The resulting residue was then subjected to silica chromatography gradient eluting with 100% petroleum ether to 40% EtOAc/petroleum ether to obtain **1** as a white solid (1.7 g, 59%). The spectra of **1** were identical to that found in literature(Walenzyk et al., 2005).

**Ethyl 8-amino-4-oxo-4H-chromene-2-carboxylate** **(2).** The nitro chromenone **1** (600 mg, 2.28 mmol) and tin (II) chloride dihydrate (1.73 g, 9.1 mmol) in 2N hydrochloric acid (5.0 mL) and EtOH (5.0 mL) were heated at 50^o^C for 3 h. 10% NaHCO_3_ solution (20 mL) and EtOAc (30 mL) was added and the solution filtered through Celite. The filtrate was then separated, and the organic layer washed with brine (30 mL). The organic layer was then dried (MgSO_4_) and concentrated *in vacuo* to give the product **2** as a yellow solid (450 mg, 85%). The spectra of **2** were identical to that found in literature (Walenzyk et al., 2005).

**General Procedure A.**

**Ethyl 4-phenethoxybenzoate** **(4).** Sodium hydride (60% mineral dispersion, 0.24 g, 6.62 mmol) was added to a stirred solution of ethyl 4-hydroxybenzoate (1 g, 6.02 mmol) and 1-bromo-2-phenylpropane (1.23 g, 6.62 mmol) in DMF (5 mL) at 20^o^C. The solution was then warmed to 50^o^C and stirred for 24 h. The solution was quenched with 1N HCl and extracted with Et_2_O (2 x 15 mL). The organic layer was washed with water, dried (MgSO_4_) and concentrated *in vacuo* to give a liquid. The liquid was subjected to silica column chromatography gradient eluting with 100% petroleum ether to 25% EtOAc/ petroleum ether to obtain **4** as a colourless liquid (0.47 g, 29%) (In this instance, it was noted that this reaction did not go to completion by TLC, so the reaction yield was compromised). ^1^H NMR (CDCl_3_): δ 8.02 (d, 2H, *J* 8.9 Hz), 7.38-7.32 (m, 5H), 6.95 (d, 2H, *J* 8.9 Hz), 4.39 (q, 2H, *J* 7.2 Hz), 4.27 (t, 2H, *J* 7.1 Hz), 3.17 (t, 2H, *J* 7.1 Hz), 1.42 (t, 3H, *J* 7.1 Hz).

**Ethyl 4-(3-phenylpropoxy)benzoate** **(5).** General Procedure A was followed using ethyl 4-hydroxybenzoate (1.00 g, 6.02 mmol) and 1-bromo-3-phenylpropane (1.32 g, 6.62 mmol), to obtain **5** as a colourless liquid (1.60 g, 94%). ^1^H NMR (CDCl_3_): δ 7.98 (2H, d, *J* 9.0 Hz), 7.29-7.20 (5H, m), 6.89 (2H, d, *J* 9.0 Hz), 4.34 (2H, q, *J* 7.1 Hz), 4.01 (2H, t, *J* 6.3 Hz), 2.82 (2H, t, *J* 7.2 Hz), 2.18-2.08 (2H, m), 1.38 (3H, t, *J* 7.1 Hz).

**Ethyl 4-(4-phenylbutoxy)benzoate** **(6).** General Procedure A was followed using ethyl 4-hydroxybenzoate (0.50 g, 3.01 mmol) and 1-bromo-4-phenylbutane (0.71 g, 3.31 mmol), to obtain **6** as a colourless liquid (0.63 g, 70%). ^1^H NMR (CDCl_3_): δ 8.04 (2H, d, *J* 9.0 Hz), 7.37-7.24 (5H, m), 6.94 (2H, d, *J* 9.0 Hz), 4.39 (2H, q, *J* 7.1 Hz), 4.05 (2H, t, *J* 6.1 Hz), 2.75 (3H, t, *J* 7.1 Hz), 1.90-1.86 (4H, m), 1.43 (3H, t, *J* 7.1 Hz).

**General Procedure B**

**4-Phenethoxybenzoic acid (8).** The ester **4** (0.4 g, 1.48 mmol) in a mixture of a 2N NaOH solution (15 mL), THF (15 mL) and EtOH (15 mL) was allowed to stir at 50^o^C for 16 h. The solution was cooled and concentrated to half the original volume. The solution was acidified with 2N hydrochloric acid solution (20 mL) and the precipitate that formed was filtered off, washing with water. The filter cake was dried in a vacuum oven and gave **8** as a white solid (340 mg, 95 %). ^1^H NMR (d_6_-DMSO): δ 7.89 (2H, d, *J* 8.7 Hz), 7.36-7.23 (5H, m), 7.06 (2H, d, *J* 8.8 Hz), 4.29 (2H, t, *J* 6.8 Hz), 2.85 (2H, t, *J* 6.8 Hz). *m/z* = 241 [M-H]^-^.

**4-(3-Phenylpropoxy)benzoic acid** **(9).** General Procedure B was followed using the ester **5** (1.25 g, 4.4 mmol) to obtain **9** as a white solid (1.13 g, 89%). ^1^H NMR (CDCl_3_): δ 8.09 (2H, d, *J* 9.0 Hz), 7.32-7.21 (5H, m), 6.94 (2H, d, *J* 9.0 Hz), 4.05 (2H, t, *J* 6.3 Hz), 2.85 (2H, t, *J* 7.2 Hz), 2.20-2.12 (2H, m). *m/z* = 255 [M-H]^-^.

**4-(4-Phenylbutoxy)benzoic acid** **(10).** General Procedure B was followed using the ester **6** (0.6 g, 2.01 mmol) to obtain **10** as a white solid (0.53 g, 98%). ^1^H NMR (d_6_-DMSO): δ 7.89 (2H, d, *J* 8.9 Hz), 7.33-7.20 (5H, m), 7.01 (2H, d, *J* 8.9 Hz), 4.08 (2H, t, *J* 6.0 Hz), 2.67 (2H, t, *J* 7.0 Hz), 1.82-1.70 (4H, m). *m/z* = 269 [M-H]^-^.

**General Procedure C.**

**Ethyl 8-(4-methoxybenzamido)-4-oxo-4H-chromene-2-carboxylate** **(20).** 4-Methoxybenzoic acid **19** (39 mg, 0.26 mmol) and thionyl chloride (25 μL, 0.34 mmol) in DCE (2 mL) was heated to 60^o^C for 2 h. The solution was then concentrated to dryness. To resulting residue was added a solution of the 8-amino chromenone **2** (20 mg, 0.09 mmol) and DIPEA (47.8 μL, 0.27 mmol) in DCE (4 mL). The solution was allowed to stir for 2 d at 20^o^C. EtOH (3 mL) was added and the precipitate was filtered off to give **20** as a white solid (20 mg, 64%). The spectra of **20** were identical to that found in literature(Funke et al., 2013).

**Ethyl 8-(4-(benzyloxy)benzamido)-4-oxo-4H-chromene-2-carboxylate** **(11).** General Procedure C was followed using 4-benzyloxybenzoic acid **7** (58.72 mg, 0.26 mmol) and the 8-amino chromenone **2** (20 mg, 0.09 mmol) to yield **11** as a white solid (38 mg, 92%). ^1^H NMR (d_6_-DMSO): δ 10.10 (1H, s), 8.07 (1H, dd, *J* 8.0 and 1.3 Hz), 7.99 (2H, d, *J* 8.7 Hz), 7.88 (2H, dd, *J* 8.1 and 1.3 Hz) 7.57-7.33 (6H, m), 7.17 (2H, d, *J* 8.8 Hz), 6.97 (1H, s), 5.22 (1H, s), 4.31 (2H, q, *J* 7.0 Hz), 1.21 (3H, t, *J* 6.8 Hz). *m/z* = 444 [M+H]^+^.

**Ethyl 4-oxo-8-(4-phenethoxybenzamido)-4H-chromene-2-carboxylate** **(12).** General Procedure C was followed using the carboxylic acid **8** (62 mg, 0.26 mmol) and the 8-amino chromenone **2** (20 mg, 0.09 mmol) to give **12** as a white solid (30 mg, 77%). ^1^H NMR (d_6_-DMSO): δ 10.09 (1H, s), 8.07 (1H, dd, *J* 8.0 and 1.3 Hz), 7.99 (2H, d, *J* 8.3 Hz), 7.87 (2H, dd, *J* 8.0 and 1.6 Hz) 7.54 (1H, t, *J* 7.9 Hz), 7.35-7.22 (5H, m), 7.09 (2H, d, *J* 8.4 Hz), 6.97 (1H, s), 4.35-4.27 (4H, m), 3.07 (2H, t, *J* 7.0 Hz), 1.22 (3H, t, *J* 7.8 Hz). *m/z* = 458 [M+H]^+^.

**Ethyl 4-oxo-8-(4-(3-phenylpropoxy)benzamido)-4H-chromene-2-carboxylate** **(13).** General Procedure C was followed using the carboxylic acid **9** (66 mg, 0.26 mmol) and the 8-amino chromenone **2** (20 mg, 0.09 mmol) to obtain **13** as a white solid (30 mg, 74%). ^1^H NMR (d_6_-DMSO): δ 10.09 (1H, s), 8.07 (1H, dd, *J* 7.7 and 1.6 Hz), 7.98 (2H, d, *J* 6.8 Hz), 7.88 (2H, dd, *J* 8.0 and 1.6 Hz) 7.54 (1H, t, *J* 7.9 Hz), 7.31-7.17 (5H, m), 7.08 (2H, d, *J* 6.9 Hz), 6.97 (1H, s), 4.32 (2H, q, *J* 7.1 Hz), 4.06 (2H, t, *J* 6.4 Hz), 2.76 (2H, t, *J* 7.2 Hz), 2.09-2.00 (2H, m), 1.22 (3H, t, *J* 7.1 Hz). *m/z* = 472 [M+H]^+^.

**Ethyl 4-oxo-8-(4-(4-phenylbutoxy)benzamido)-4H-chromene-2-carboxylate** **(14).** General Procedure C was followed using the carboxylic acid **10** (70 mg, 0.26 mmol) and the 8-amino chromenone **2** (20 mg, 0.09 mmol) to obtain **14** as a white solid (33 mg, 79%). ^1^H NMR (d_6_-DMSO): δ 10.10 (1H, s), 8.09 (1H, dd, *J* 7.8 and 1.6 Hz), 8.00 (2H, d, *J* 6.8 Hz), 7.88 (2H, dd, *J* 8.0 and 1.6 Hz), 7.57 (1H, t, *J* 7.9 Hz), 7.33-7.18 (5H, m), 7.09 (2H, d, *J* 8.9 Hz), 7.00 (1H, s), 4.33 (2H, q, *J* 7.1 Hz), 4.13-4.08 (2H, m), 2.67 (2H, t, *J* 7.3 Hz), 1.78-1.74 (4H, m), 1.24 (3H, t, *J* 7.1 Hz). *m/z* = 486 [M+H]^+^.

**General Procedure D.**

**8-(4-Methoxybenzamido)-4-oxo-4H-chromene-2-carboxylic acid** **(21).** A solution of K_2_CO_3_ (10 mg, 0.07 mmol) in water (4.5 mL) was added to the ester **20** (20 mg, 0.05 mmol) in THF (12 mL) and EtOH (3 mL). The reaction mixture was stirred at 20^o^C for 24 h. The mixture was acidified to pH 2 with 2N hydrochloric acid. The organic solvents were removed under reduced pressure. The resulting precipitate was filtered off, washed with water, and dried in a vacuum oven. The crude material was purified from EtOH and acetone. Acetone was left to evaporate to give a precipitate that was filtered off to give **21** as a pale yellow solid (8 mg, 43%). The spectra of **21** were identical to that found in literature(Funke et al., 2013).

**8-(4-(Benzyloxy)benzamido)-4-oxo-4H-chromene-2-carboxylic acid** (**15).** General Procedure D was followed using the ester **11** (30 mg, 0.07 mmol) to obtain **15** as a white solid (5 mg, 18%). ^1^H NMR (d_6_-DMSO): δ 10.79 (1H, s), 8.06 (1H, dd, *J* 7.8 and 1.7 Hz), 8.00 (2H, d, *J* 8.9 Hz), 7.88 (1H, dd, *J* 8.0 and 1.7 Hz), 7.56-7.33 (6H, m), 7.17 (2H, d, *J* 8.9 Hz), 6.92 (1H, s), 5.21 (2H, s). *m/z* = 416 [M+H]^+^.

**4-Oxo-8-(4-phenethoxybenzamido)-4H-chromene-2-carboxylic acid** **(16).** General Procedure D was followed using the ester **8** (30 mg, 0.07 mmol) to obtain **16** as a white solid (10 mg, 35%). ^1^H NMR (d_6_-DMSO): δ 10.10 (1H, s), 8.13 (1H, dd, *J* 7.7 and 1.5 Hz), 8.00 (2H, d, *J* 8.8 Hz), 7.80 (2H, dd, *J* 8.0 and 1.5 Hz), 7.44 (1H, t, *J* 7.9 Hz), 7.36-7.22 (5H, m), 7.08 (2H, d, *J* 8.8 Hz), 6.69 (1H, s), 4.33 (2H, q, *J* 7.1 Hz), 4.30 (2H, t, *J* 6.9 Hz), 3.08 (2H, t, *J* 6.7 Hz). *m/z* = 429 [M+H]^+^.

**4-Oxo-8-(4-(3-phenylpropoxy)benzamido)-4H-chromene-2-carboxylic acid** **(17).** General Procedure D was followed using the ester **13** (40 mg, 0.08 mmol) to obtain **17** as a white solid (15 mg, 40%). ^1^H NMR (d_6_-DMSO): δ 10.07 (1H, s), 8.12 (1H, dd, *J* 7.7 and 1.5 Hz), 8.01 (2H, d, *J* 8.8 Hz), 7.86 (1H, dd, *J* 8.0 and 1.6 Hz), 7.51 (1H, t, *J* 7.9 Hz), 7.32-7.17 (5H, m), 7.08 (2H, d, *J* 8.9 Hz), 6.84 (1H, s), 4.07 (2H, t, *J* 6.4 Hz), 2.77 (2H, t, *J* 7.3 Hz), 2.11-2.01 (2H, m). *m/z* = 442 [M-H]^-^.

**4-Oxo-8-(4-(4-phenylbutoxy)benzamido)-4H-chromene-2-carboxylic acid** **(18).** General Procedure D was followed using the ester **14** (30 mg, 0.07 mmol) to obtain **18** as a white solid (10 mg, 35%). ^1^H NMR (d_6_-DMSO): δ 10.06 (1H, s), 8.06 (2H, d, *J* 7.3 Hz), 7.98 (2H, d, *J* 8.6 Hz), 7.88 (1H, dd, *J* 7.9 and 1.1 Hz), 7.56-7.51 (1H, m), 7.30-7.17 (5H, m), 7.07 (2H, d, *J* 8.6 Hz), 6.93 (1H, s), 4.09 (2H, t, *J* 5.5 Hz), 2.65 (2H, t, *J* 5.7 Hz), 1.75-1.72 (4H, m). *m/z* = 458 [M+H]^+^.

**General Procedure E.**

***N*-(3-Acetyl-2-hydroxyphenyl)-4-methoxybenzamide** **(22).** 4-Methoxybenzoic acid **19** (0.24 g, 1.59 mmol) and thionyl chloride (384 μL, 5.29 mmol) in DCE (2 mL) was heated to 60^o^C for 2 h. The solution was then concentrated to dryness. To resulting residue was added a solution of 1-(3-amino-2-hydroxy-phenyl) ethanone (0.2 g, 1.32 mmol) in DCE (2.5 mL) and pyridine (2.5 mL) at 0^o^C. The reaction mixture was stirred for 4 h at 20^o^C. The reaction was quenched with a 2N hydrochloric acid solution and extracted with EtOAc (2 x 20 mL). The organic layer was washed with 2N hydrochloric acid (20 mL) and then a 10% NaHCO_3_ solution (20 mL). The organic layer was then dried (MgSO_4_) and concentrated *in vacuo*. The resulting residue was subjected to silica chromatography gradient eluting from 100% hexanes to 30% EtOAc/hexanes to give **22** as a pale yellow solid (200 mg, 53%). ^1^H NMR (CDCl_3_): δ 8.79 (1H, dd, *J* 8.1 and 1.3 Hz), 8.61 (1H, br s), 7.91 (2H, d, *J* 8.8 Hz), 7.51 (1H, dd, *J* 8.2 and 1.5 Hz), 7.03-6.97 (3H, m), 3.90 (3H, s), 2.69 (3H, s). *m/z* = 286 [M+H]^+^.

***N*-(3-Acetyl-2-hydroxyphenyl)-4-(3-phenylpropoxy)benzamide** **(23).** General Procedure E was followed using the acid **9** (170 mg, 0.66 mmol) and 1-(3-amino-2-hydroxy-phenyl) ethanone (100 mg, 0.66 mmol) to obtain **22** as a pale yellow solid (175 mg, 68%). ^1^H NMR (CDCl_3_): δ 8.79 (1H, dd, *J* 8.0 and 1.2), 8.60 (1H, br s), 7.90 (2H, d, *J* 6.8 and 2.13 Hz), 7.51 (1H, dd, *J* 8.1 and 1.4 Hz), 7.35-7.22 (5H, m), 7.02-6.97 (3H, m), 4.05 (2H, t, *J* 6.3 Hz), 2.86 (2H, t, *J* 7.3 Hz), 2.68 (3H, s), 2.21-2.12 (2H, m). *m/z* = 389 [M+H]^+^.

**General Procedure F.**

**4-Methoxy-*N*-(4-oxo-2-(2H-tetrazol-5-yl)-4H-chromen-8-yl)benzamide** **(24).** To a stirred suspension of the acetophenone **22** (0.19 g, 0.67 mmol) and sodium butoxide (410 mg, 3.66 mmol) in anhydrous DMF (2 mL) is added a solution of ethyl 1H-tetrazole-5-carboxylate (0.12 g, 0.87 mmol) in anhydrous DMF (2 mL). The resulting mixture is heated to 50 ^o^C for 20 h. 2N hydrochloric acid was added and the mixture extracted with EtOAc (2 x 10 mL). The organic layer was washed with brine (10 mL), dried (MgSO_4_) and concentrated *in vacuo* to give a crude residue. The residue was subjected to silica chromatography gradient eluting with 100% hexanes to 40% EtOAc/hexanes to give the diketo intermediate as a pale yellow solid (115 mg). Methanol (2 mL) containing concentrated hydrochloric acid (210 μL) is added to the diketo intermediate (115 mg) and the mixture heated to reflux for 2 h. The mixture was then concentrated to half the volume and cooled on ice. The precipitate that formed was filtered off, washing with MeOH to give **24** as a pale yellow solid (55 mg, 30%). ^1^H NMR (d_6_-DMSO): δ 10.12 (1H, br s), 8.34 (1H, d, *J* 6.4 Hz), 8.04-8.00 (3H, m), 7.51 (1H, dd, *J* 8.1 and 1.4 Hz), 7.09 (2H, d, *J* 8.9 Hz), 6.40 (1H, d, *J* 6.4 Hz), 3.86 (3H, s). *m/z* = 364 [M+H]^+^.

***N*-(4-Oxo-2-(2H-tetrazol-5-yl)-4H-chromen-8-yl)-4-(3-phenylpropoxy)benzamide** **(25).** General Procedure F was followed using the acetophenone **23** (190 mg, 0.67 mmol) and ethyl 1H-tetrazole-5-carboxylate (120 mg, 0.87 mmol) to obtain **25** as a pale yellow solid (20 mg, 17%). ^1^H NMR (d_6_-DMSO): δ 10.06 (1H, br s), 8.25 (1H, d, *J* 6.4 Hz), 8.06-8.03 (2H, m), 7.92-7.90 (1H, m), 7.56 (1H, t, J 7.9 Hz), 7.34-7.20 (7H, m), 7.11 (2H, d, *J* 7.1 Hz), 4.15-4.05 (2H, m), 2.81-2.72 (2H, m), 2.12-2.03 (2H, m). *m/z* = 468 [M+H]^+^.

**References**

Funke, M., Thimm, D., Schiedel, A.C., Muller, C.E., 2013. 8-Benzamidochromen-4-one-2-carboxylic acids: potent and selective agonists for the orphan G protein-coupled receptor GPR35. J Med Chem 56, 5182-5197.

Walenzyk, T., Carola, C., Buchholz, H., König, B., 2005. Chromone derivatives which bind to human hair. Tetrahedron 61, 7366-7377.

**^1^H-NMR and LCMS of final compounds**

Ethyl 8-(4-methoxybenzamido)-4-oxo-4H-chromene-2-carboxylate **20**

***^1^H-NMR (CDCl_3_)***


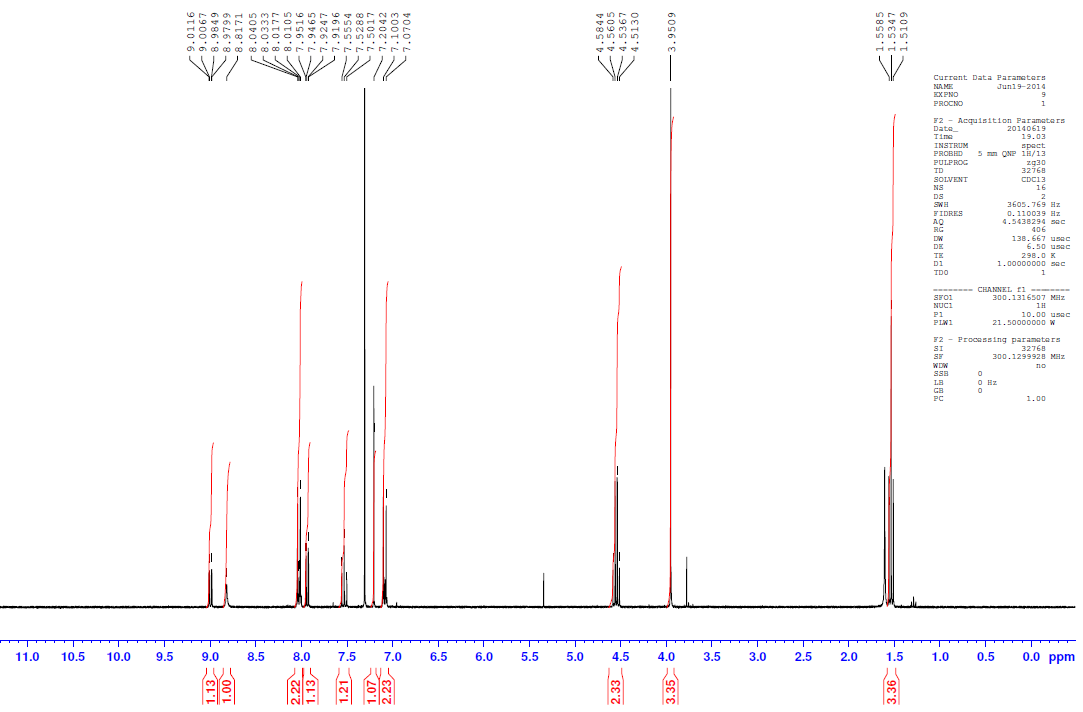


***LCMS (254 nm)***


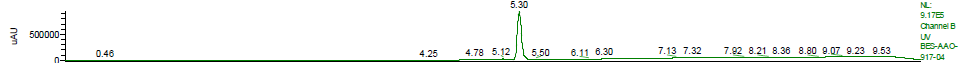


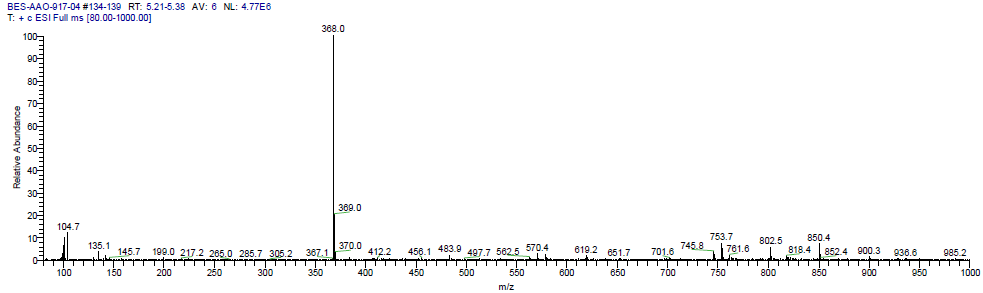


Ethyl 8-(4-(benzyloxy)benzamido)-4-oxo-4H-chromene-2-carboxylate **11**

***^1^H-NMR (d_6_-DMSO)***


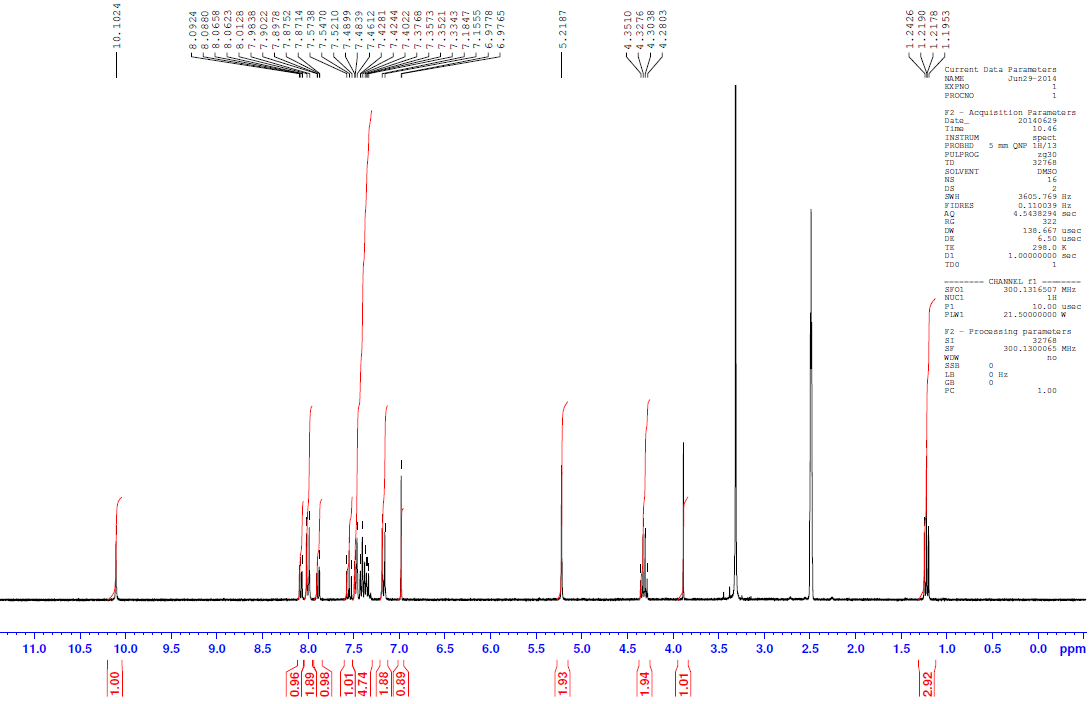


***LCMS (254 nm)***


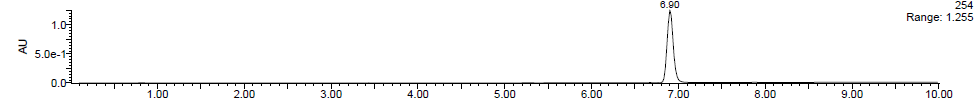


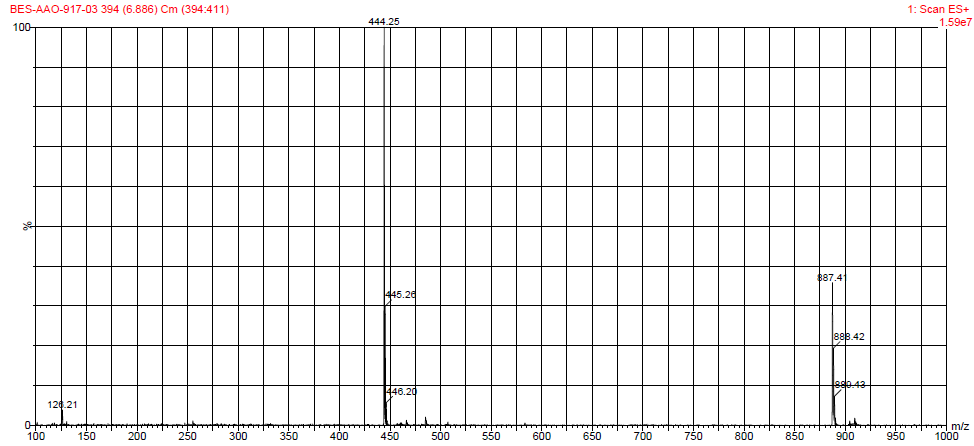


Ethyl 4-oxo-8-(4-phenethoxybenzamido)-4H-chromene-2-carboxylate **12**

***^1^H-NMR (d_6_-DMSO)***


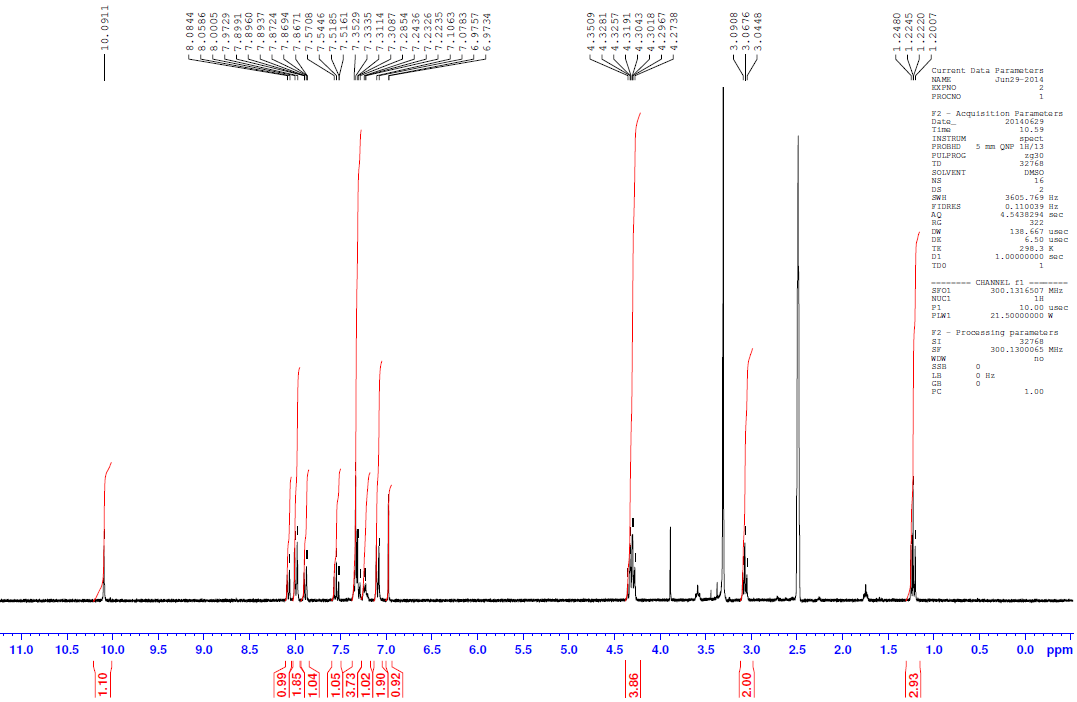


***LCMS (254 nm)***


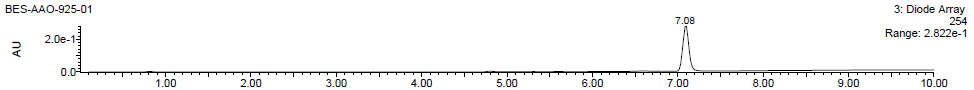


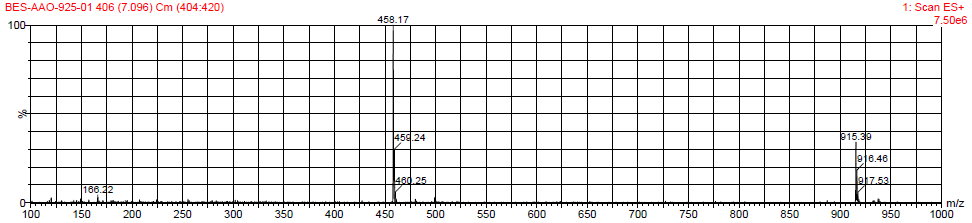


Ethyl 4-oxo-8-(4-(3-phenylpropoxy)benzamido)-4H-chromene-2-carboxylate **13**

***^1^H-NMR (d_6_-DMSO)***


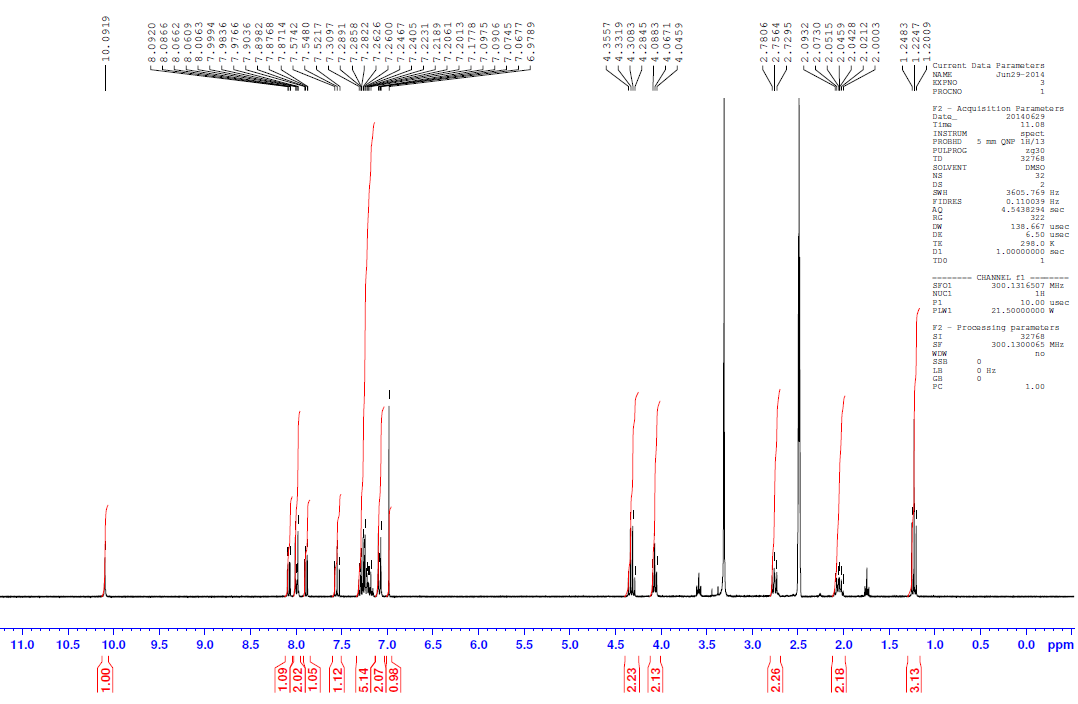


***LCMS (254 nm)***


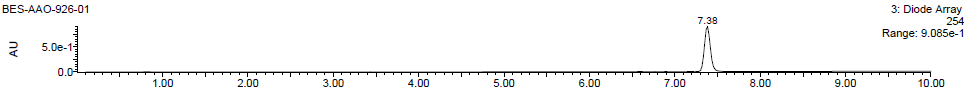


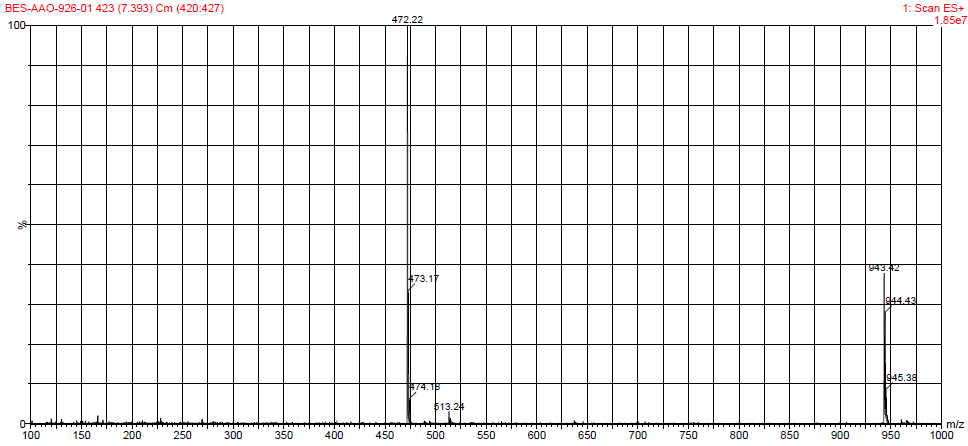


Ethyl 4-oxo-8-(4-(4-phenylbutoxy)benzamido)-4H-chromene-2-carboxylate **14**

***^1^H-NMR (d_6_-DMSO)***


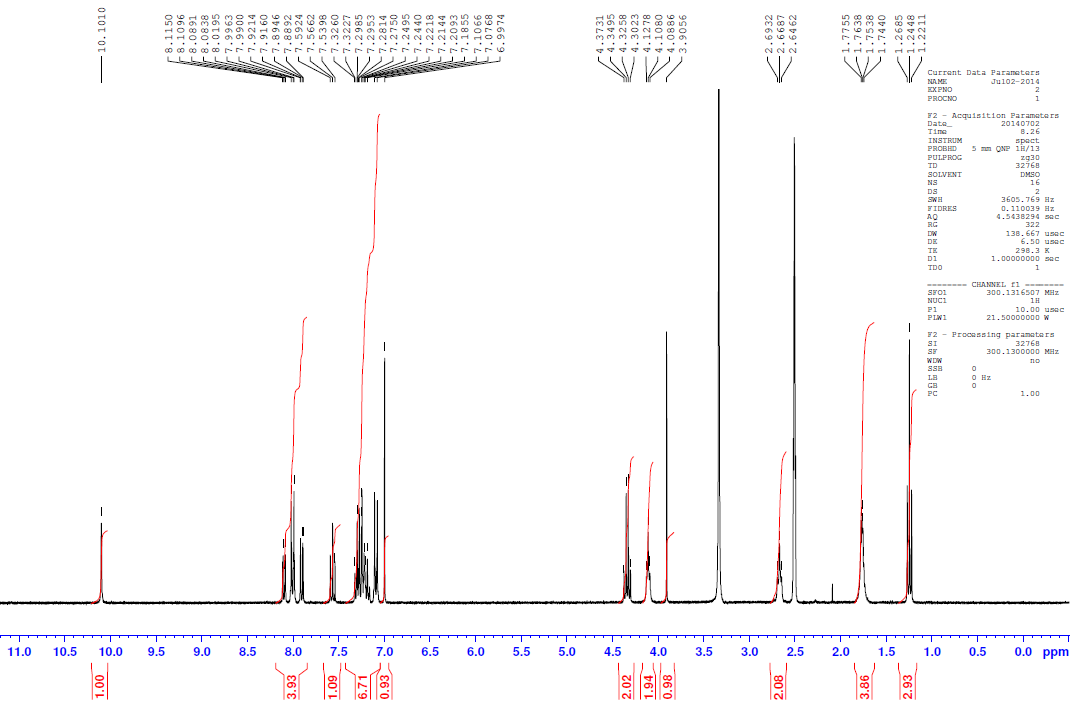


***LCMS (254 nm)***


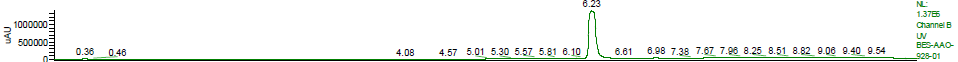


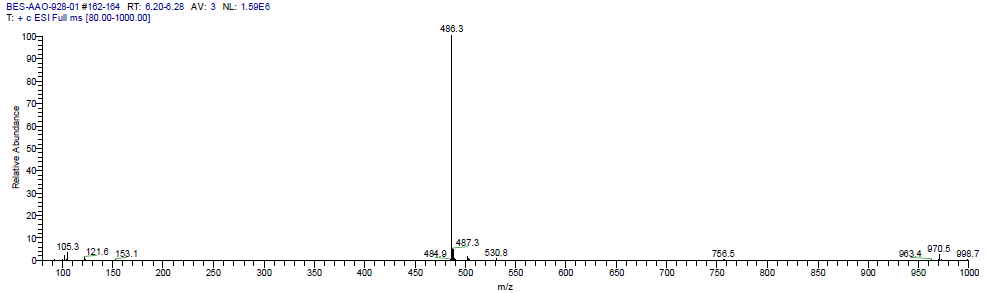


8-(4-Methoxybenzamido)-4-oxo-4H-chromene-2-carboxylic acid **21**

***^1^H-NMR (d_6_-DMSO)***


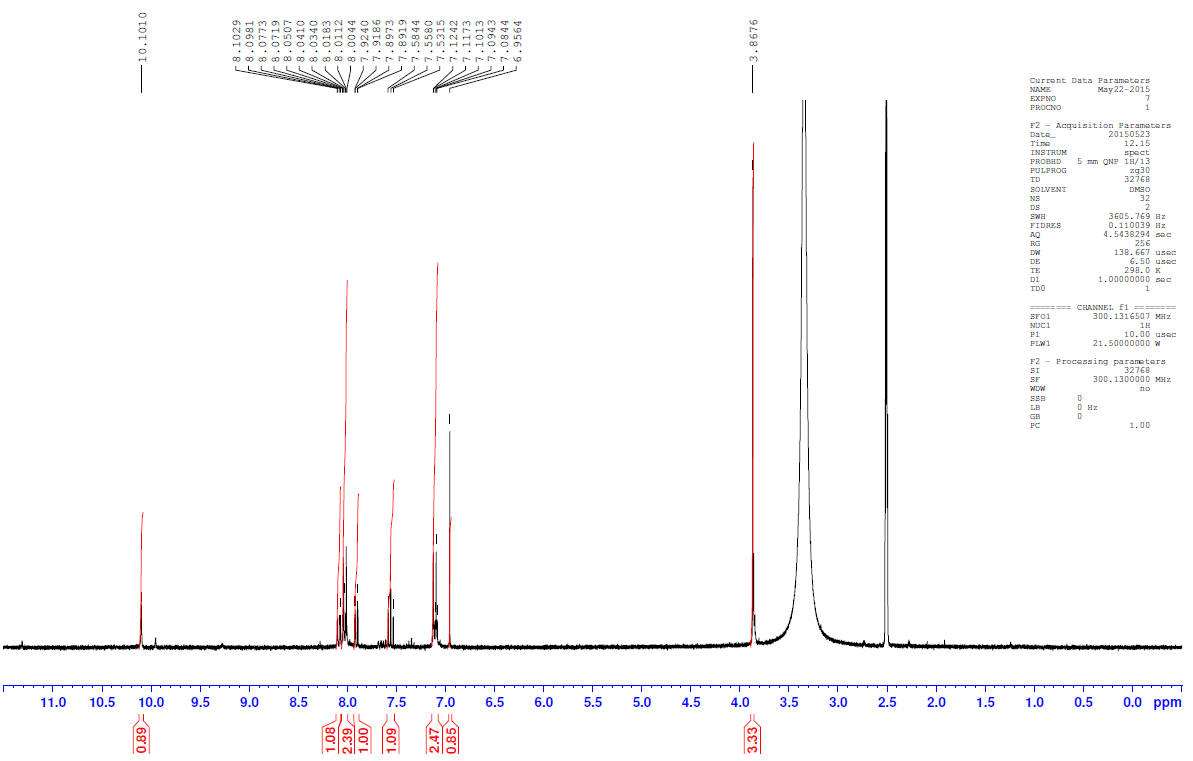


***LCMS (254 nm)***


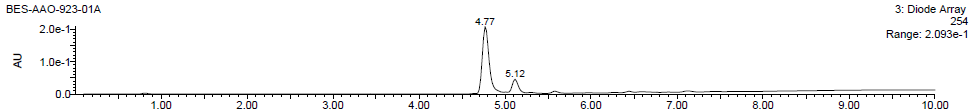


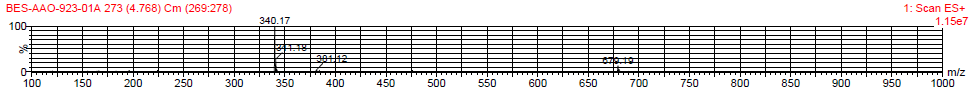


8-(4-(Benzyloxy)benzamido)-4-oxo-4H-chromene-2-carboxylic acid **15**

***^1^H-NMR (d_6_-DMSO)***


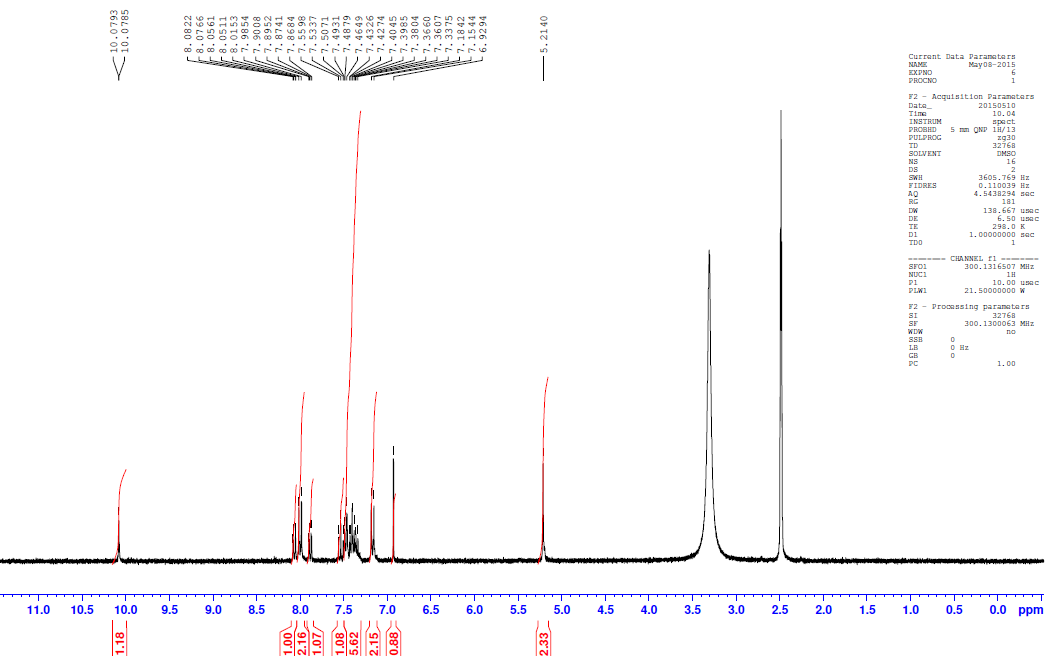


***LCMS (254 nm)***


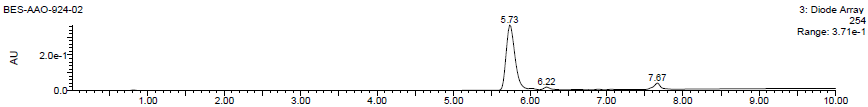


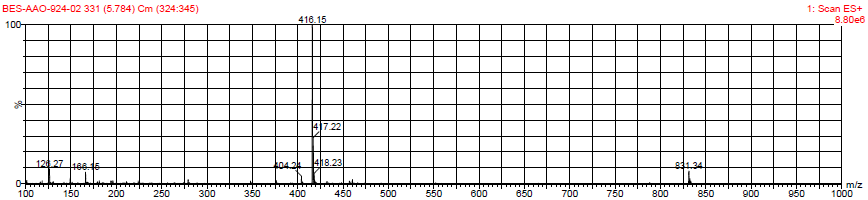


4-Oxo-8-(4-phenethoxybenzamido)-4H-chromene-2-carboxylic acid **16**

***^1^H-NMR (d_6_-DMSO)***


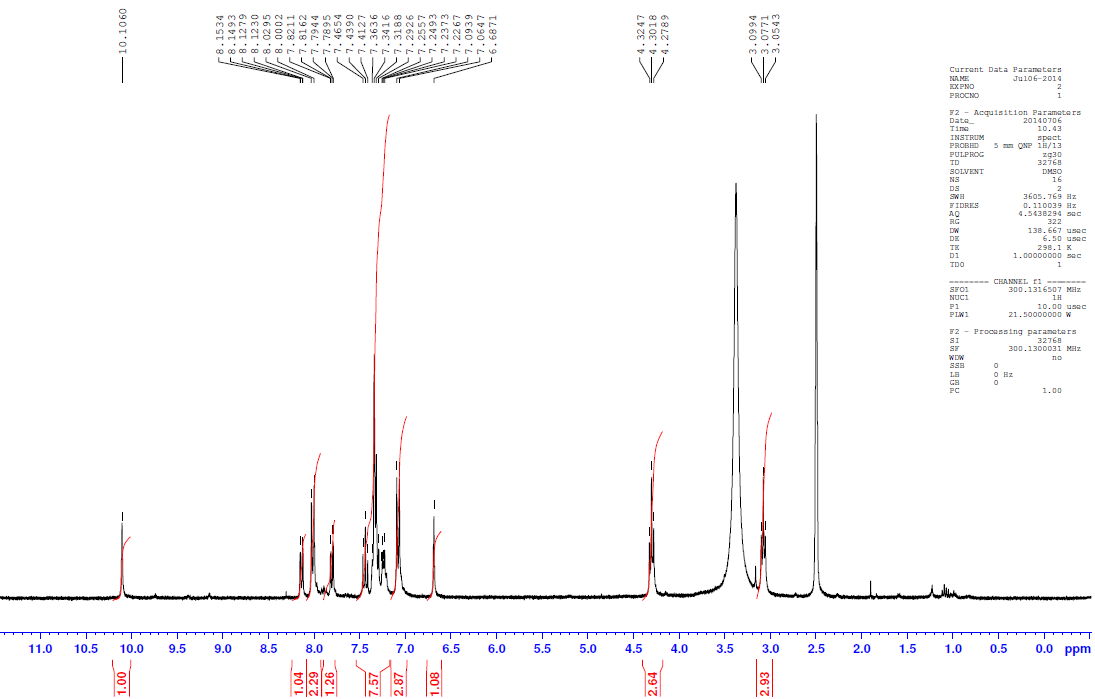


***LCMS (254 nm)***


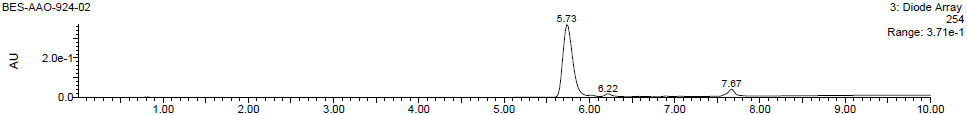


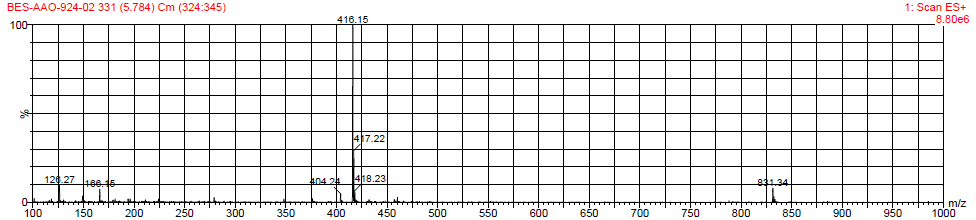


4-Oxo-8-(4-(3-phenylpropoxy)benzamido)-4H-chromene-2-carboxylic acid **17**

***^1^H-NMR (d_6_-DMSO)***


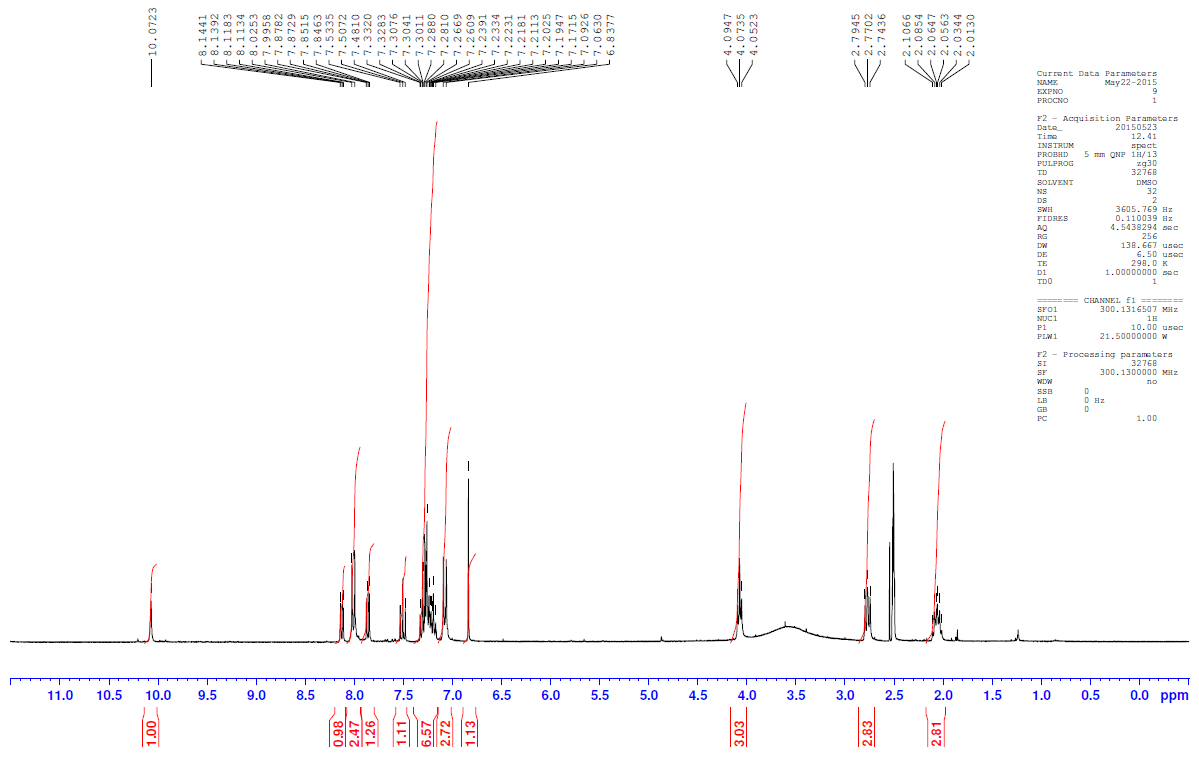


***LCMS (254 nm)***


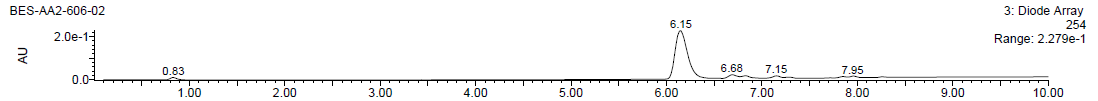


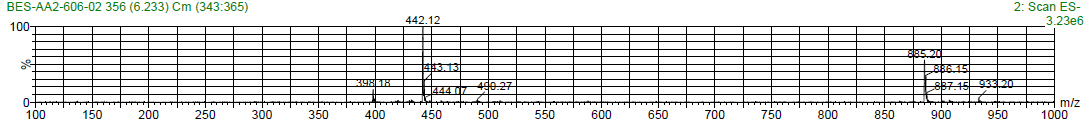


4-Oxo-8-(4-(4-phenylbutoxy)benzamido)-4H-chromene-2-carboxylic acid **18**

***^1^H-NMR (d_6_-DMSO)***


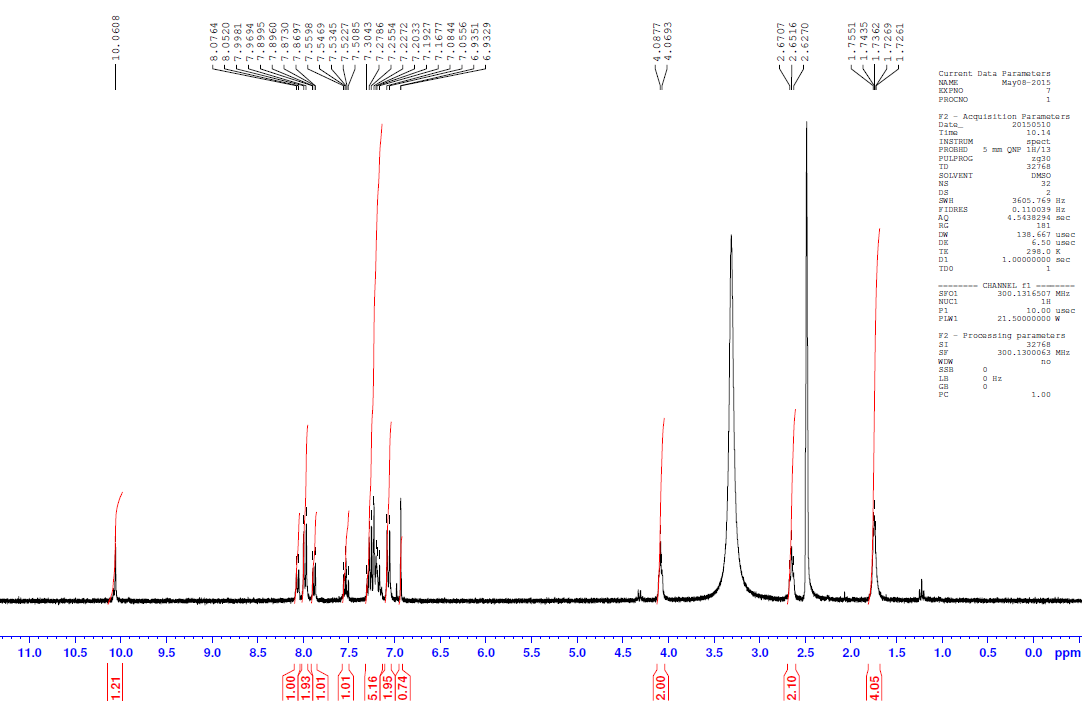


***LCMS (254 nm)***


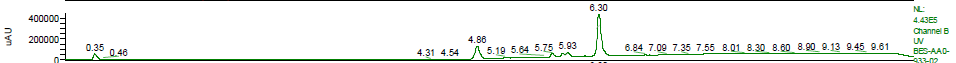


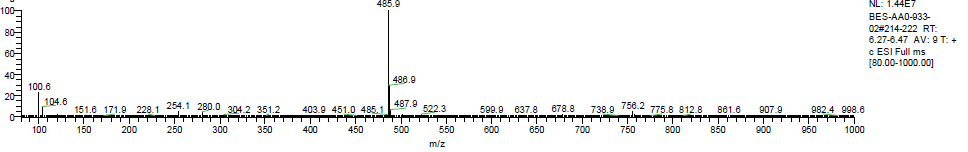


4-Methoxy-*N*-(4-oxo-2-(2H-tetrazol-5-yl)-4H-chromen-8-yl)benzamide **24**

***^1^H-NMR (d_6_-DMSO)***


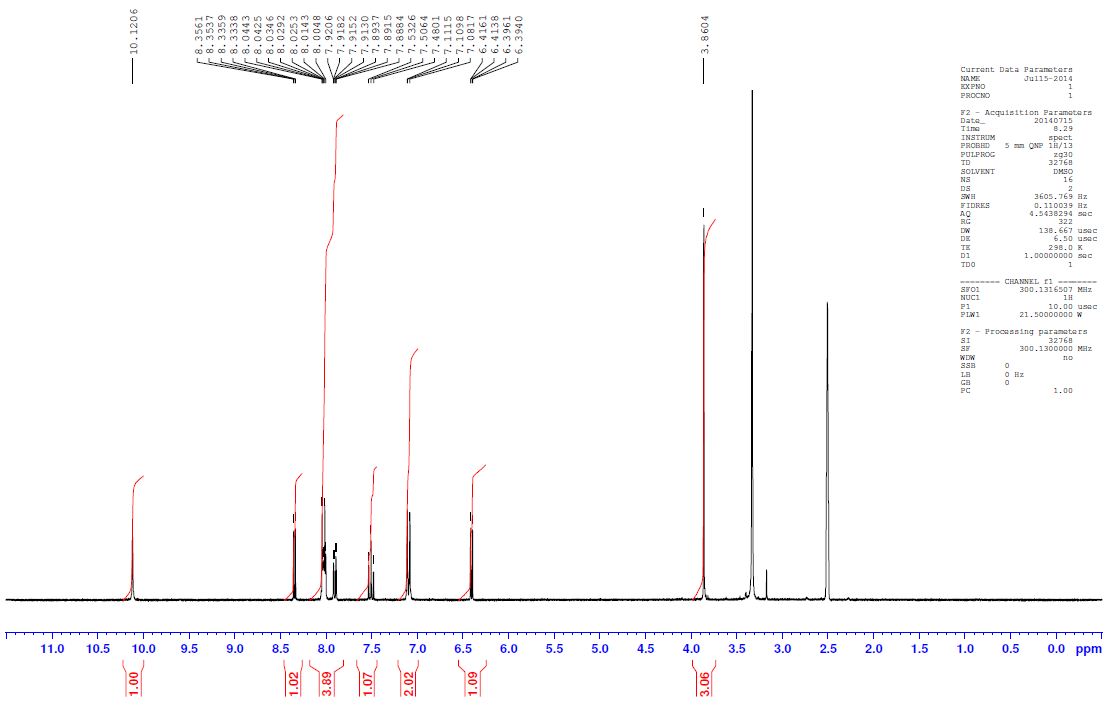


***LCMS (254 nm)***


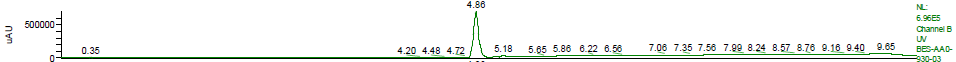


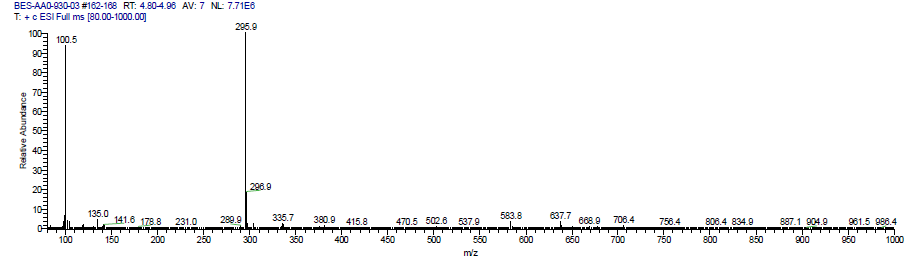


*N*-(4-Oxo-2-(2H-tetrazol-5-yl)-4H-chromen-8-yl)-4-(3-phenylpropoxy)benzamide **25**

***^1^H-NMR (d_6_-DMSO)***


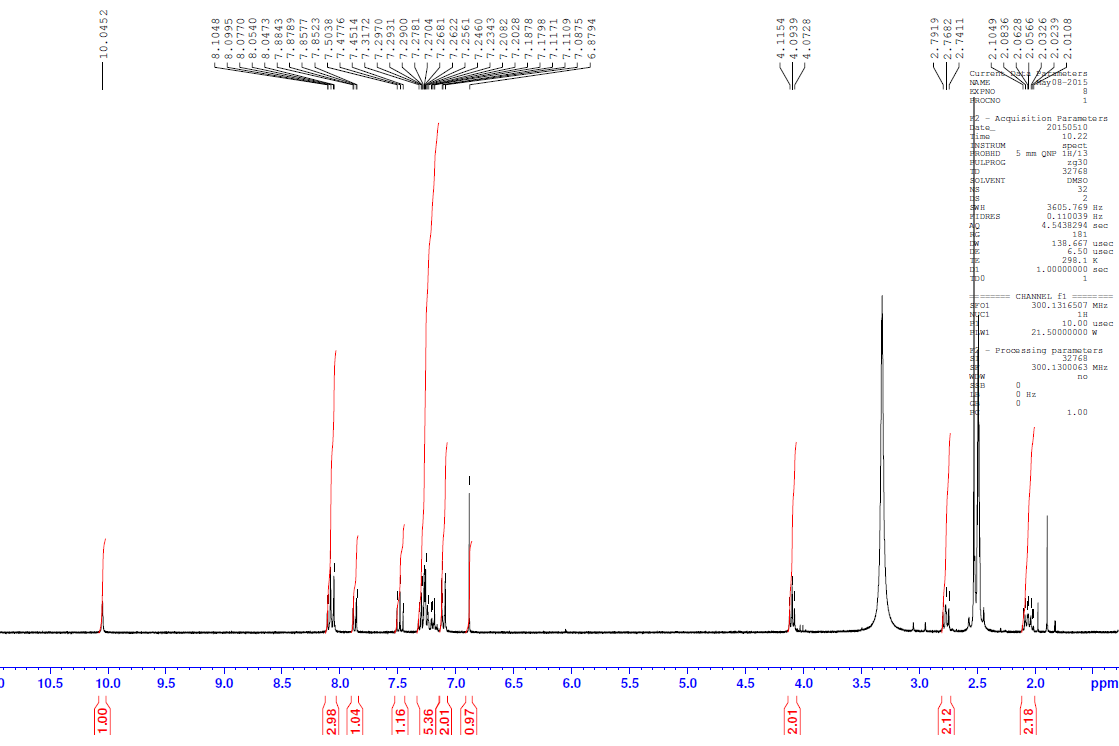


***LCMS (254 nm)***


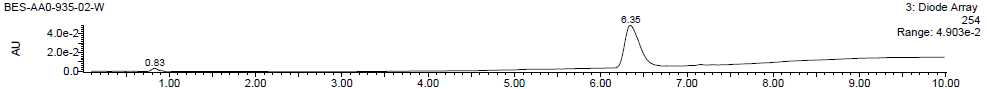


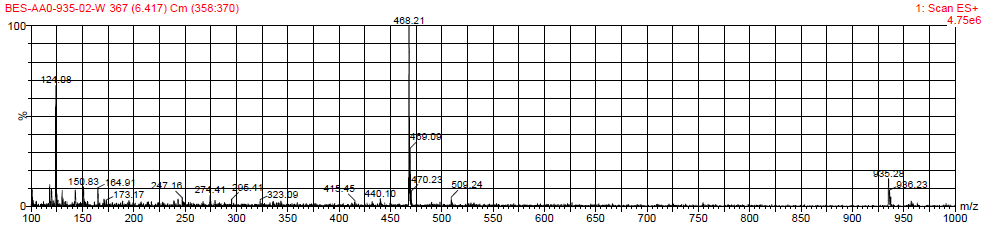

Supplement: Multimedia component 1 [file mmc1.docx]
